# Complexes of Neutralizing and Non-Neutralizing Affinity Matured Fabs with a Mimetic of the Internal Trimeric Coiled-Coil of HIV-1 gp41

**DOI:** 10.1371/journal.pone.0078187

**Published:** 2013-11-07

**Authors:** Elena Gustchina, Mi Li, Rodolfo Ghirlando, Peter Schuck, John M. Louis, Jason Pierson, Prashant Rao, Sriram Subramaniam, Alla Gustchina, G. Marius Clore, Alexander Wlodawer

**Affiliations:** 1 Laboratory of Chemical Physics, National Institute of Diabetes and Digestive and Kidney Diseases, National Institutes of Health, Bethesda, Maryland, United States of America; 2 Macromolecular Crystallography Laboratory, National Cancer Institute, Frederick, Maryland, United States of America; 3 Basic Research Program, SAIC-Frederick, Frederick, Maryland, United States of America; 4 Laboratory of Molecular Biology, National Institute of Diabetes and Digestive and Kidney Diseases, National Institutes of Health, Bethesda, Maryland, United States of America; 5 Laboratory of Cellular Imaging and Macromolecular Biophysics, National Institute of Biomedical Imaging and Bioengineering, National Institutes of Health, Bethesda, Maryland, United States of America; 6 FEI Company, Hillsboro, Oregon, United States of America; 7 Laboratory of Cell Biology, Center for Cancer Research, National Cancer Institute, Bethesda, Maryland, United States of America; University of Queensland, Australia

## Abstract

A series of mini-antibodies (monovalent and bivalent Fabs) targeting the conserved internal trimeric coiled-coil of the N-heptad repeat (N-HR) of HIV-1 gp41 has been previously constructed and reported. Crystal structures of two closely related monovalent Fabs, one (Fab 8066) broadly neutralizing across a wide panel of HIV-1 subtype B and C viruses, and the other (Fab 8062) non-neutralizing, representing the extremes of this series, were previously solved as complexes with 5-Helix, a gp41 pre-hairpin intermediate mimetic. Binding of these Fabs to covalently stabilized chimeric trimers of N-peptides of HIV-1 gp41 (named (CCIZN36)_3_ or 3-H) has now been investigated using X-ray crystallography, cryo-electron microscopy, and a variety of biophysical methods. Crystal structures of the complexes between 3-H and Fab 8066 and Fab 8062 were determined at 2.8 and 3.0 Å resolution, respectively. Although the structures of the complexes with the neutralizing Fab 8066 and its non-neutralizing counterpart Fab 8062 were generally similar, small differences between them could be correlated with the biological properties of these antibodies. The conformations of the corresponding CDRs of each antibody in the complexes with 3-H and 5-Helix are very similar. The adaptation to a different target upon complex formation is predominantly achieved by changes in the structure of the trimer of N-HR helices, as well as by adjustment of the orientation of the Fab molecule relative to the N-HR in the complex, via rigid-body movement. The structural data presented here indicate that binding of three Fabs 8062 with high affinity requires more significant changes in the structure of the N-HR trimer compared to binding of Fab 8066. A comparative analysis of the structures of Fabs complexed to different gp41 intermediate mimetics allows further evaluation of biological relevance for generation of neutralizing antibodies, as well as provides novel structural insights into immunogen design.

## Introduction

The envelope of HIV-1 and, particularly, its most conserved region, the transmembrane protein gp41 which mediates membrane fusion during viral entry into the host cell, has been for decades a target of extensive efforts involving structural and biophysical research, drug design, and immunogen design. Nevertheless, new properties of gp41 are still being discovered through continuing studies. The membrane proximal region (MPER) of gp41, as well as the N- and C-terminal helices are highly conserved regions within the HIV envelope glycoprotein, which is formed by trimerization of the gp120-gp41 heterodimer. Following CD4 and co-receptor binding to gp120, there are structural changes in gp41 that ultimately lead to membrane fusion, making gp41 an attractive target for immunogen design. A number of antibodies directed to the gp41 region have been discovered and their binding to gp41 has been characterized using X-ray crystallography [1]–[3] and cryo-electron microscopy [4], [5]. It has been shown that the potency of Fabs and/or scFvs was generally higher than of the corresponding complete antibody molecules, suggesting that crowding around the epitope might impose spacial constraints [3].

In previous studies [6],[7] we characterized a series of broadly neutralizing mini-antibodies (monovalent and bivalent Fabs) derived from the HuCAL GOLD synthetic human combinatorial antibody library [8], comprising more than 10^10^ human specificities, by panning against the chimeric HIV-1 gp41-derived construct N_CCG_-gp41 [9]. It is of interest to note that the heavy chain of the originally selected Fab is encoded by the master gene that corresponds to the sequence of the VH1-69 gene shared by the D5 antibody [1] isolated from the naïve human B-cell library (VH1-69*01) and the HK-20 antibody [3] derived from an immortalized memory B cell (VH1-69*05) of an HIV-1-infected individual. These three antibodies, although derived from unrelated sources using different selection procedures, were found to be directed against the same conformational epitope that includes a hydrophobic pocket on the N terminal helix of gp41. This VH gene was also found to be preferentially used in the immune responses directed against coreceptor-binding site of HIV gp120, as well as HCV E2 [10]. The initially identified parental Fab 3674 was subjected to affinity maturation against the N_CCG_-gp41 antigen using targeted diversification of the CDR-H2 loop. This procedure resulted in significant enhancement of HIV-1 neutralization properties of the new antibodies, both in terms of potency and neutralization breadth, over standardized panels of envelope glycoproteins (Envs) from contemporary primary isolates of HIV-1 subtypes B and C [7]. However, in some instances the neutralization properties of very closely related Fabs varied widely and were not necessarily correlated with their affinity to the target antigen [7].

We previously reported crystal structures of the complexes of two Fabs representing the extremes of this series in terms of their neutralization properties with a chimeric protein 5-Helix [11], derived from the ectodomain of HIV-1 gp41 [2]. One of the antibodies, Fab 8066, was broadly neutralizing, while the other, Fab 8062, was non-neutralizing. These two Fabs were structurally almost identical, differing only in four residues located in their CDR H2 (I53L, T56F, T57A, and N58V for 8066 and 8062, respectively). Although both Fabs 8066 and 8062 are able to bind to the exposed trimer of N-helices with similarly high affinity [7], their ability to bind to the 5-Helix construct differs by two orders of magnitude [2], and that difference is correlated with their neutralizing activity. Analysis of the structural data suggests that, as a consequence of spatial and steric restraints, the internal N-HR trimeric coiled-coil of gp41 may never be fully exposed during HIV-1 entry, or that the six-helix bundle may be formed sequentially in a step-wise manner, and that the key to neutralization lies in the ability of the Fab or antibody to compete with binding of C-HR helices, required to form the six-helix bundle of gp41. However, all modeling performed by us [2] or by others [3] was based on experimentally derived structures that contained only a single Fab molecule bound to 5-Helix, thus any interactions between multiple antibody molecules bound to their targets could only be assumed.

In order to extrapolate the dynamics of the antibody/antigen complex formation and to enhance our understanding by structural insights into the intrinsic properties of the antibody/antigen complexes, a family of covalently stabilized chimeric N-peptides was chosen for further structural studies and potential future rounds of affinity maturation [12], [13]. Purification was optimized for the (CCIZN36)_3_ peptide (called 3-H throughout this paper) that exposes, in a stable manner, the complete N-HR internal trimeric coiled coil of gp41. 3-H is a disulfide-linked trimer that includes three copies of the N-HR helix of gp41. In this study we describe the structures of the complexes of 3-H with Fabs 8066 and 8062 in which one molecule of 3-H was bound to three Fabs, as well as biophysical properties of such complexes.

## Results

To examine the binding of Fabs 8066 and 8062 to an exposed trimer of N-helices, an expression construct for the stabilized N-HR trimer mimetic 3-H was designed and the peptide was expressed and purified. Characterization of the binding of three Fab molecules to the 3-H, as well as investigation of the properties of the resulting complexes, were carried out using a combination of biophysical and structural techniques.

### Expression and Purification of the Chimeric Peptide, CCIZN36

The chimeric peptide CCIZN36 uses a isoleucine zipper (IZ; [14]) to aid in the formation and stabilization of a trimeric coiled coil of gp41 N-HR helices. A comparison of the numbering scheme utilized previously and in this work is shown in Table S1 in File SI. The expression vector was constructed essentially as described using the thioredoxin protein as a fusion partner to aid expression and disulfide bridge formation [15]. Fusion protein was expressed and purified by affinity chromatography, followed by thrombin cleavage and elution of the disulfide-linked 3-H. The resulting peptide was further purified by reverse-phase chromatography and molecular weight and disulfide formation was verified by mass-spectrometry and SDS gel electrophoresis (see Methods, Fig. S1 in File SI).

### The gp41 Mimetic (CCIZN36)_3_ (3-H) and Fabs are Monodisperse

**Figure 1:**
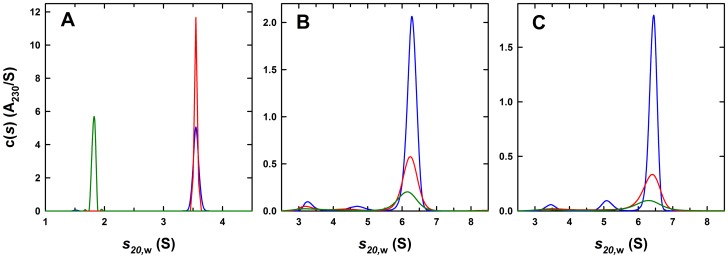
Characterization of the 3-H, the Fab 8066 and Fab 8062 and their complexes by analytical ultracentrifugation. (A) Sedimentation *c(s)* distributions for the 3-H at 6 µM (green), the Fab 8062 at 1.4 µM (blue) and the Fab 8066 at 1.7 µM (red). (B) Sedimentation *c(s)* distributions for a purified (Fab 8062)_3_/3-H complex at loading concentrations of 0.66 (blue), 0.33 (red) and 0.15 µM (green). (C) Sedimentation *c(s)* distributions for a purified (Fab 8066)_3_/3-H complex at loading concentrations of 0.4 (blue), 0.2 (red) and 0.1 µM (green). All data are based on sedimentation velocity data collected at 50 krpm and 25.0°C using the absorbance optical system at 230 nm.

Sedimentation velocity experiments were carried out to characterize the various 3-H and antibody preparations. *C(s)* sedimentation profiles for different preparations of the 3-H at concentrations of 2–6 µM showed the presence of a single species at 1.82±0.02 S having an estimated molar mass of 22.0±2.5 kDa, consistent with the presence of a cross-linked trimer (M_calc_ = 22,683 Da) (Fig. 1A). Based on the integrated interference signal obtained from sedimentation experiments, we noted that spectrophotometric absorbances at 280 nm were not always a reliable indicator of the true 3-H concentration. This is most likely due to the adsorption of 3-H on the surface of the cuvette, and when possible we used concentrations inferred from the sedimentation experiments. Samples of the 8062 and 8066 Fabs at 1.4 and 1.7 µM, respectively, showed the presence of a single, monodisperse species consistent with a Fab monomer. Fab 8062 had a sedimentation coefficient of 3.56±0.01 S and an estimated molar mass of 46±4 kDa (M_calc_ = 48,879 Da), whereas the Fab 8066 had a sedimentation coefficient of 3.55±0.01 S and a mass of 49±2 kDa (M_calc_ = 48,878 Da) (Fig. 1A).

### 3-H Forms a High Affinity Complex with Three Fab Molecules

To determine the stoichiometry of 3-H to the two Fabs, sedimentation experiments were carried out on the purified complexes formed with Fabs 8062 and 8066. In both cases, a single complex in which three Fabs were bound to a single 3-H was observed. For Fab 8062, the complex was characterized by a sedimentation coefficient of 6.27±0.06 S and an estimated molar mass of 146±15 kDa (M_calc_ = 169,321 Da) (Fig. 1B). No evidence for complex dissociation was observed in the concentration range studied (0.15–1.5 µM), indicative of a high affinity interaction. Similarly, in the case of Fab 8066, a single complex at 6.38±0.06 S with a molar mass of 150±7 kDa was observed, consistent with the binding of three antibody molecules (M_calc_ = 169,318 Da) (Fig. 1C). As in the case of the complex formed with Fab 8062, no significant dissociation was observed in the studied concentration range of 0.1–1.0 µM.

### Evidence for the Lack of Cooperativity

**Figure 2:**
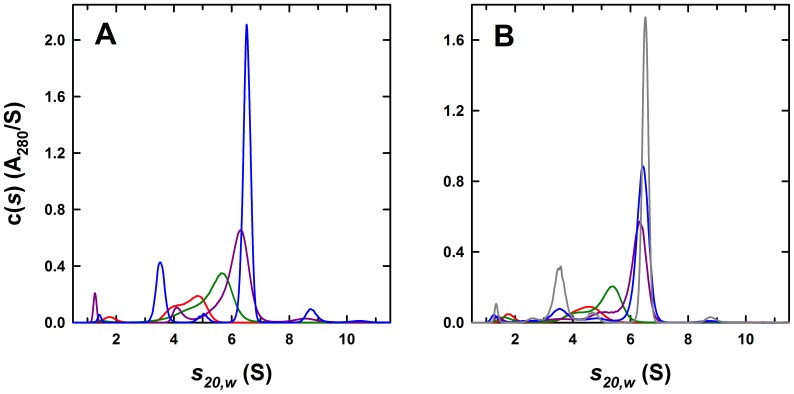
Fab 8066 and 8062 titrations. (A) Sedimentation *c(s)* distributions for samples containing 2.4 µM of the 3-H and 2.1 (red), 4.3 (green), 6.4 (purple) and 8.5 µM (blue) of added Fab 8066. (B) Sedimentation *c(s)* distributions for samples containing 2.0 µM of the 3-H and 1.2 (red), 2.4 (green), 4.8 (purple), 6.0 (blue) and 7.2 µM (gray) of added Fab 8062. In both cases the actual amount of CCIZN36 was estimated from the integral contribution of the complex at saturation. Profiles presented are based on sedimentation velocity data collected at 50 krpm and 25.0°C using the absorbance optical system at 280 nm. Similar profiles were observed using the interference optics.

**Figure 3:**
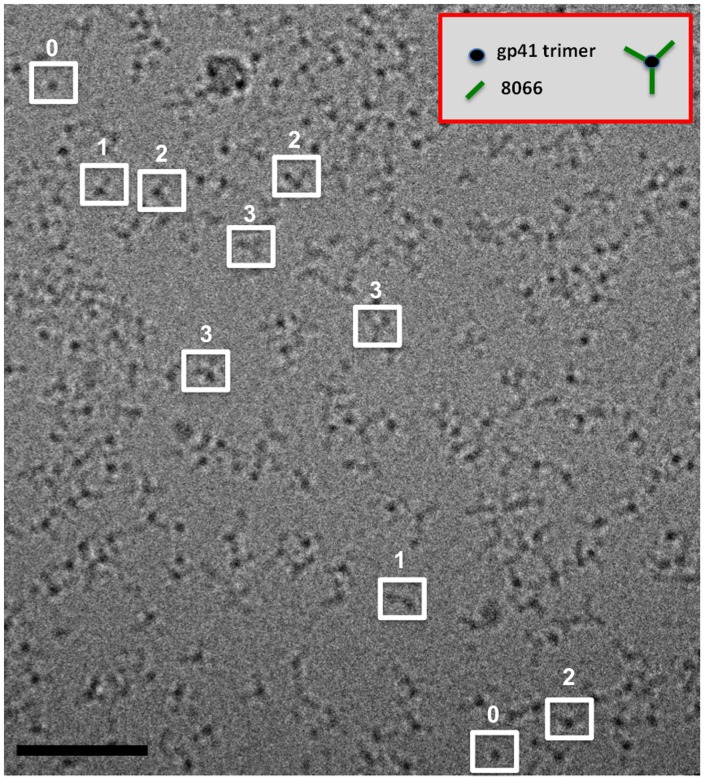
Projection cryo-electron microscopic image from specimens of gp41-Fab 8066. The complexes were vitrified at a concentration of ∼0.2 mg/ml. Examples of putative complexes with Fab occupancies of 0, 1, 2, or 3 are highlighted in the boxed regions. Data were collected on an FEI Titan Krios electron microscope, operated at 80 kV, with the images recorded on a Falcon II direct electron detector. The black bar corresponds to 50 nm.

To ascertain whether the interaction of the three Fab molecules with the 3-H shows any form of positive or negative cooperativity, a series of sedimentation velocity experiments were carried out with increasing amounts of Fabs added to a solution of the 3-H. The *c(s)* profiles obtained show the population of intermediate species with complete formation of the saturated trimer-antibody 6.53 S complex only at stoichiometric amounts of antibody (1 trimer :3 antibodies), illustrating the absence of any strong positive or negative cooperativity of binding (Fig. 2). Analysis of Fab 8066/3-H complexes by cryo-electron microscopy establishes the presence of complexes with 0, 1, 2 or 3 bound Fab molecules, confirming the results from the sedimentation experiments (Fig. 3). These data further support a 3∶1 Fab to 3-H stoichiometry as the complete complex is observed with three equivalents of antibody. These data are also consistent with a high affinity interaction as free antibody is only observed beyond saturation. Based on these observations and subsequent global modeling (see below), we conclude that antibody binding occurs without any strong positive or negative cooperativity.

### Determination of the Binding Affinity

**Figure 4:**
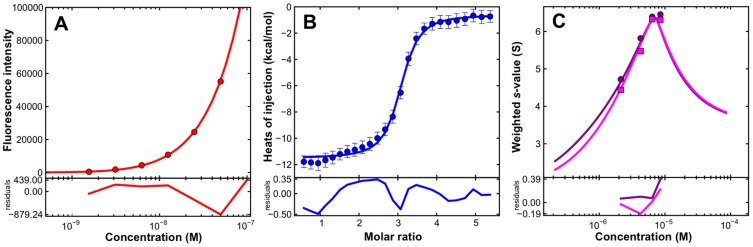
The interaction of the Fab 8066 with 3-H. Solid lines show the global best-fit to a non-cooperative A+B+B+B interaction in which the trimer A has three symmetric binding sites. A global analysis of the data results in a K_d1_ affinity of 4.3 nM. (A) Binding affinity of Fab 8066 to the 3-H. Corrected fluorescence intensity differences (325–355 nm) as a function of the complex concentration in a dilution experiment using a (Fab 8066)_3_/3-H trimer complex previously characterized by sedimentation velocity (Fig. X1C). (B) Binding enthalpy of Fab 8066 to the 3-H. Isothermal titration calorimetry data for the titration of 19 µM Fab 8066 into a solution containing 1.77 µM trimer. (C) Interference *s_w_* isotherm (magenta) and absorbance *s_w_* (purple) isotherm (Fig. 2A) data along with the best-fits obtained in a global analysis of the data sets shown.

**Figure 5:**
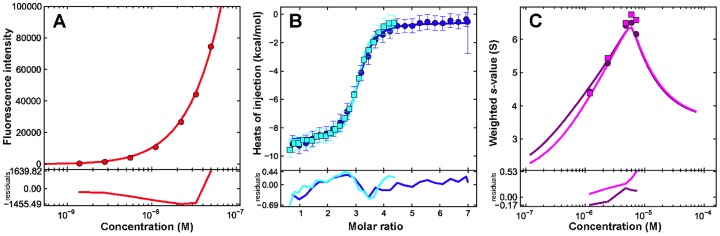
The interaction of the Fab 8062 with (CCIZN36)_3_. Solid lines show the global best-fit to a non-cooperative A+B+B+B interaction in which the trimer A has three symmetric binding sites. A global analysis of the data returns a K_d1_ affinity of 4.2 nM. (A) Binding affinity of 8062 for the 3-H. Corrected fluorescence intensity differences (325–355 nm) as a function of the complex concentration in a dilution experiment using a Fab 8062/3-H complex previously characterized by sedimentation velocity (Fig. X1B). (B) Binding enthalpy of 8062 for the 3-H. Isothermal titration calorimetry data for the titration of 18 µM 8062 into a solution containing 0.67 µM (blue) or 1.00 µM (cyan) trimer. (C) Interference *s_w_* isotherm (magenta) and absorbance *s_w_* (purple) isotherm (Fig. 2B) data along with the best-fits obtained in a global analysis of the data sets shown.

To determine the binding affinity of the Fabs for the 3-H, experiments were carried out at a concentration range spanning the expected affinity of interaction, utilizing the intrinsic tryptophan fluorescence provided by the unlabeled 3-H and the Fabs. Signal differences between emissions at 325 and 355 nm were exploited in order to maximize the information provided through the observed blue shift. Dilutions of the purified complexes were carried out and data were analyzed in terms of a non-cooperative A+B+B+B interaction. An analysis of these dilution experiments results in essentially identical binding affinities (Figs. 4A, 5A, Fig. S2 in File SI). In the case of the Fab 8062 complex a K_d1_ of 10.0±2.0 nM was obtained, whereas in the case of the 8066 antibody complex data were best-fit with a K_d1_ of 5.0±1.0 nM.

### Determination of the Binding Enthalpy

ITC experiments were carried out to determine the binding enthalpies. The results were analyzed in SEDPHAT utilizing the binding affinities obtained from the fluorescence dilution data. As reliable heats of injection could only be obtained at low micromolar concentration of 3-H, these experiments are essentially carried out in a regime where stoichiometric binding occurs, providing for a good measure of the binding enthalpy. Even though we observe stoichiometric binding, the isotherms still show significant curvature, reflecting the 9-fold range of macroscopic affinities (K_d1_, K_d2_ and K_d3_) in the absence of any cooperativity. The global analysis of two ITC experiments for the titration of Fab 8062 leads to a binding enthalpy ΔH(AB) of −9.2±0.2 kcal mol^−1^ (Fig. 5B). These fits require that only 82–85% of the expected trimer concentration be active, once more highlighting the previously noted problems with reliably determining the concentration of the 3-H. A titration of the Fab 8066 leads to a binding enthalpy ΔH(AB) of −11.1±0.2 kcal mol^−1^ (Fig. 4B), but this necessitates that only 42% of the trimer be active for binding.

### Global Data Analysis

To reconcile the observations made by sedimentation velocity with the fluorescence dilution and ITC experiments, weighted average sedimentation coefficient (*s_w_*) isotherms based on data shown in Fig. 2 were constructed and analyzed globally. In the case of Fab 8066, a global analysis of the ITC, fluorescence and sedimentation data results in a K_d1_ affinity of 4.3 nM (68% confidence interval of 2.0–7.9 nM) with a reasonably good fit (Fig. 4). Similarly, in the case of Fab 8062, a global analysis results in an indistinguishable K_d1_ affinity of 4.2 nM (68% confidence interval of 1.1–10.2 nM) (Fig. 5). Sedimentation coefficients for the trimer (1.82 S, Fig. 1A), Fab (3.55 S, Fig. 1A) and ABBB complex (6.53 S, Fig. 2A) were fixed in these analyses.

In summary, the sedimentation velocity, ITC and fluorescence experiments support a high affinity interaction of Fabs 8062 and 8066 with the 3-H. Based on the global analysis presented, which brings together independent observables from calorimetric, fluorescence and sedimentation experiments [16], there is no evidence for either a strong positive or strong negative cooperativity of binding, although one cannot definitively rule out weak cooperative effects that would be difficult to define using current methods.

### Crystal Structures of the Complexes of Fabs 8066 and 8062 with 3-H

**Figure 6:**
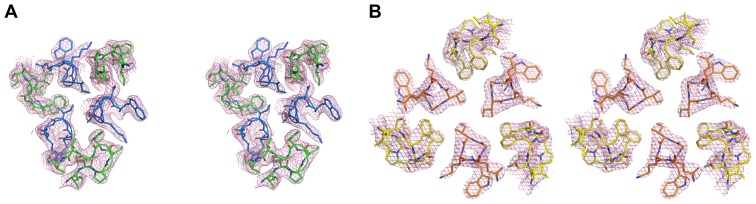
Electron density for the fragments of the structure comprising CDRs H2 of the Fabs and N helices of 3-H. The 2Fo-Fc maps are contoured at 1.0 σ and are shown for the fragments of N helices comprising residues 571–575, and for CDRs H2 (residues 51–57). A) (Fab 8066)_3_/3-H. Fab is green and 3-H is blue. B) (Fab 8062)_3_/3-H. Fab is yellow and 3-H is orange.

**Figure 7:**
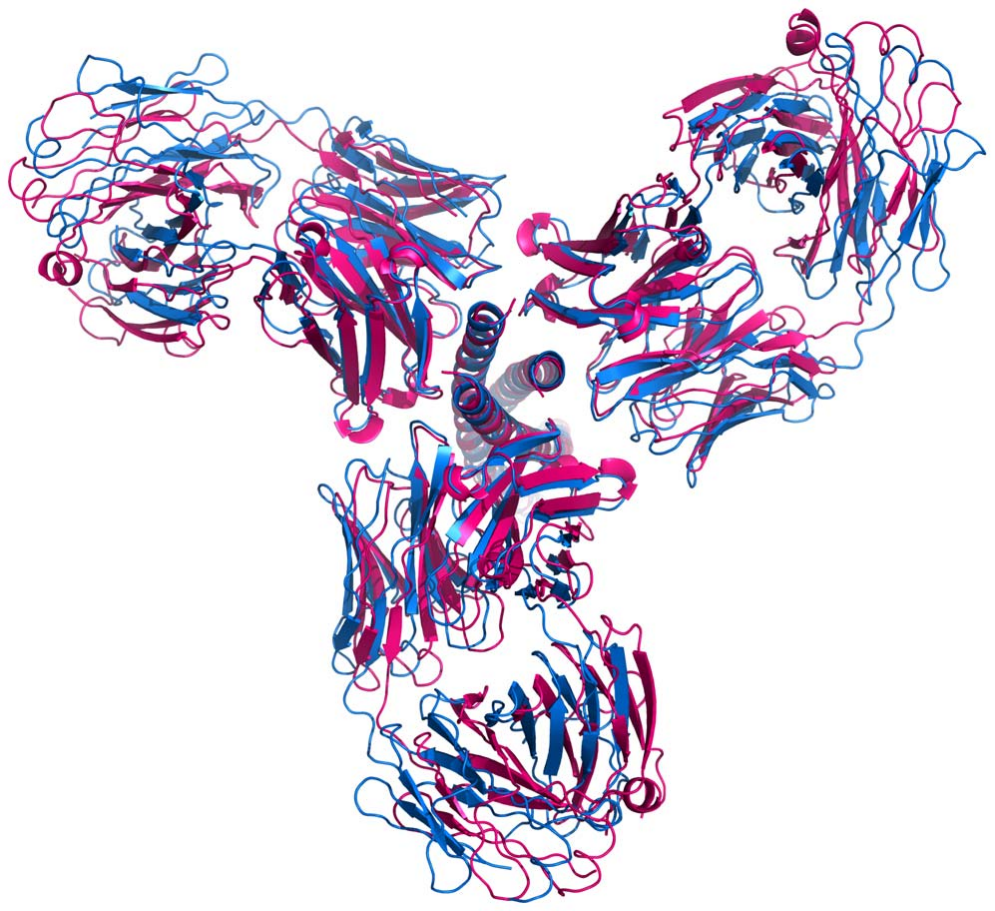
Overall view of the superimposed (Fab)_3_/3-H complexes. Each complex used for superposition was generated by the application of its own crystal symmetry and only the Cα coordinates of the helices of 3-H were superimposed. The (Fab 8066)_3_/3-H complex is shown in red and the (Fab 8062)_3_/3-H complex in blue.

The crystal structures of Fabs 8066 and 8062 complexed to 3-H were determined at 2.8 and 3.0 Å resolution, respectively (Table 1). Examples of the electron density for the fragments comprising CDRs H2 and N-HR helices in both complexes are shown in Fig. 6. Both Fabs bind to 3-H with a molar ratio 3∶1. Although 3-H is a single molecule consisting of three helices covalently linked with disulfide bridges, the asymmetric unit of the crystal contained only one of these helices and a single Fab. The 3-H molecule is located on a three-fold symmetry axis of the crystal, creating a crystallographic trimer consisting of complete 3-H and three Fabs. An overall view of these complexes superimposed on the basis of the Cα coordinates of the helices of 3-H is shown in Fig. 7. The epitope on CCIZN36 that is recognized by both antibodies is generally the same as was described in the complexes with 5-Helix [2]. In the crystals of both complexes the 3-H molecules are packed in a head-to-tail fashion, forming extended helical rods (Fig. S3A in File SI) with perfect hydrogen bonding between the main chain atoms of the first helical turn of one molecule and the last helical turn of the next one (Fig. S3B in File SI). Although formation of infinite helical rods in crystals has been previously noted when molecules containing long helices had been crystallized [17], such continuity of the interactions is not very common.

**Table 1:** Crystallographic data collection and refinement.

	H3/Fab 8066	H3/Fab 8062
**Data collection**		
Space group	*P*321	*P*321
Molecules/a.u.	1 Fab, 1 helix of H-3	1 Fab, 1 helix of H-3
Unit cell *a* = *b*, *c* (Å)	104.4, 104.4, 97.1	106.8, 106.8, 98.5
Resolution (Å)*	50.0-2.8 (2.9-2.8)	50.0-3.0 (3.05-3.0)
*R*_merge_ †	6.3 (32.8)	9.4 (41.0)
No. of reflections (measured/unique)	101,080/14,235	88,257/13,242
<*I*/σ*I*>	23.4 (2.1)	20.5 (1.6)
Completeness (%)	94.6 (62.7)	94.4 (63.7)
Redundancy	7.3 (2.0)	6.1 (1.8)
**Refinement**		
Resolution (Å)	35.56-2.82	30.95-3.0
No. of reflections (refinement/*R*_free_)	14,212/716	13,236/663
*R*/*R*_free_ ‡	21.46/26.56	23.66/28.95
Matthews coeff. V_m_	2.71	2.88
B factors (Å^2^)		
CCIZN	134.3	131.0
Fab	134.6	103.1
No. atoms		
Protein	3686	3625
Ligand/ion/water	0	0
R.m.s.d. from ideality		
Bond lengths (Å)	0.010	0.004
Bond angles (°)	1.34	0.80
PDB code	4KHT	4KHX

* The highest resolution shell is shown in parentheses.

† *R*_merge_ = ∑_h_∑_i_|*I*_i_-〈*I*〉|/∑_h_∑_i_ *I*_i_, where I_i_ is the observed intensity of the i-th measurement of reflection h, and 〈I〉 is the average intensity of that reflection obtained from multiple observations.

‡ *R* = ∑∥*F_o_*|-|*F_c_*∥/∑*|F_o_|*, where F_o_ and F_c_ are the observed and calculated structure factors, respectively, calculated for all data. *R*_free_ was defined in ref. [43].

### Comparison of the Complexes of Fab 8062 and 8066 with 3-H

**Figure 8:**
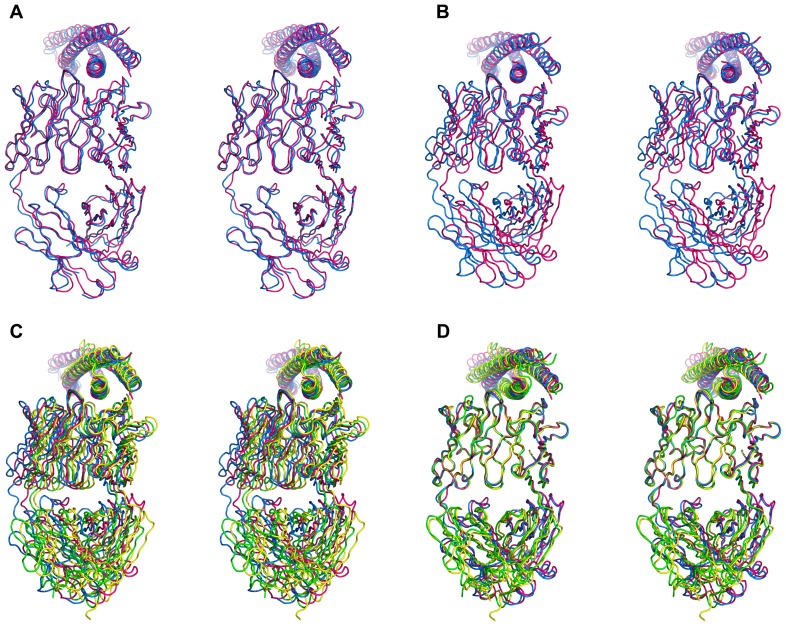
Different superpositions of the complexes of two Fabs with 3-H and 5-Helix. (A) Overall superposition of the complexes was generated by superimposing the Cα coordinates of 3 Fabs and 3-H with the program Align. For clarity, only a single Fab of each complex is shown (Fab 8066/3-Hcomplex in red, Fab 8062/3-H complex in blue). (B) Superposition as in A, but based on the Cα atoms of 3-H. (C) A ribbon diagram of (Fab 8066)_3_/3-H complex (shown in red), (Fab 8062)_3_/3-H complex (shown in blue), Fab 8066/5-Helix complex (shown in green) and Fab 8062/5-Helix complex (shown in yellow) superimposed based on Cα coordinates of 3 N-HR helices. (D) Superposition of single Fabs in the complexes with 3-H and 5-Helix based on Cα atoms of the β-sheet framework of variable domains. (Fab 8066)_3_/3-H complex is red, (Fab 8062)_3_/3-Hcomplex is blue, Fab 8066/5-Helix complex is green, and Fab 8062/5-Helix complex is yellow. Only the N-HR helices of 5-Helix are shown.

A comparative analysis of the structures of the complexes of Fabs 8066 and 8062 with 3-H is complicated by the fact that the individual differences in the interactions of a single antibody with the antigen may propagate into the secondary layer of variations in the structures of the trimer of the complexes upon its formation, through interactions between crystallographically related molecules. A straightforward approach to compare the structures by performing an overall superimposition of the two complexes using all Cα atoms (with programs such as Align [18] or SSM [19] yielded very inconclusive results (Fig. 8A; for clarity, only a single Fab molecule is shown), because all differences were averaged for both counterparts and spread between 3-H and the Fabs. In a superposition with Align the maximum Cα-Cα distance is 2.8 Å and the rms deviation is 1.2 Å for 1407 Cα atom pairs. We found that more interpretable data could be obtained when a comparative analysis of the structures described here, as well as a comparison with the previously determined structures of the same antibodies complexed to 5-Helix ([2]; PDB codes 3MA9 and 3MAC, respectively), are performed by addressing the individual components of the complexes separately.

Superposition of two complexes based on Cα coordinates of 3-H molecules does not lead to accurate superposition of the antibodies (Fig. 8B). Such a phenomenon was previously observed in the complexes of Fabs 8066 and 8062 with 5-Helix (Fig. 3b–d in Ref. [2]). That observation suggests that, upon complex formation, structural differences in the CDR H2 of the two antibodies induce changes in their orientation towards both 3-H and 5-Helix. Altogether, the respective Fab molecules in these four complexes were rotated relative to each other (Fig. 8C), and such a rotation angle in reality defines the differences in the mutual orientation of two axes: the pseudo 2-fold axis relating the VL and VH chains of the variable domain, and the three-fold symmetry axis of the 3-H molecule in the crystals of both complexes. The extent of rotation was evaluated by selecting the variable domains of the Fabs of the complexes and superimposing them with the program SSM [19], embedded within Coot [20]). The rotation angle was subsequently extracted from the rotation matrix. The rotation angle between the variable domains of Fab 8066 in the complexes with 5-Helix and 3-H was 8.0°, whereas the respective angle for Fab 8062 was 10.1°. In the complexes with 3-H the rotation angle between the variable domains of Fabs 8062 and 8066 was 5.5°.

The differences in the orientation of two antibody molecules can be corrected by applying a rigid body shift to the Fab 8062 β-sheet framework of a single molecule (HL) in the position relative to the β-sheet framework of a corresponding Fab 8066 molecule in the complexes with 3-H. That approach leads to very accurate overall superposition of the variable domains of one Fab out of three per complex (Fig. S4 in File SI). The figure also shows that, as expected, the other two Fabs are not accurately superimposed, and neither are the helices of 3-H. When the structures of Fabs 8066 and 8062 in the complexes with 5-Helix are brought into the same orientation, more significant differences in the structure of the three N-HR helices of the 5-Helix construct are revealed (Fig. 8D). The latter figure also shows substantial variations in the elbow angles of the antibodies in the four complexes. The elbow angles (defined as the angle between the pseudo 2-fold axes relating VL to VH of the variable domain, and CL to CH1 of the constant domain [21]) of the 8066 and 8062 Fabs complexed with 5-Helix and 3-H were calculated using the *elbow_angle* script of the program PyMol [22]. In the complexes with 5-Helix [2] these angles were 169° for Fab 8066 and 166° for Fab 8062. The elbow angles were very significantly different in the complexes with 3-H (123° and 126°, respectively). Superposition based on β-sheet frameworks of the antibodies also amplifies the fine variations in the conformation of respective CDRs in two complexes, in a manner described by [23].

**Figure 9:**
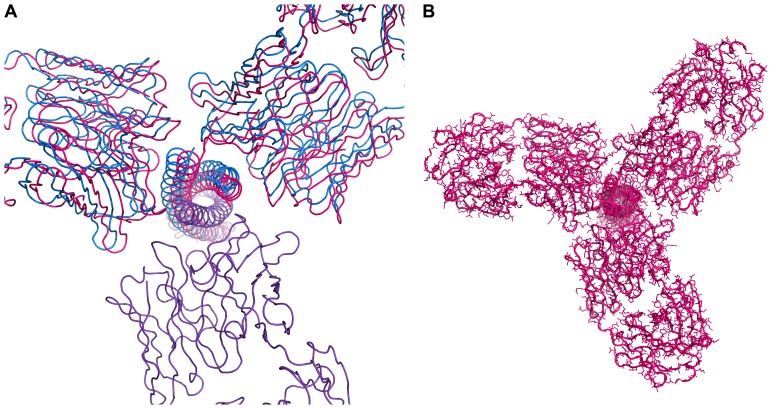
Different orientation of the Fab β-sheet frameworks in (Fab 8062)_3_/3-H complex in relation to the 3-fold axis of symmetry of 3-H are obligatory. (A) A single Fab of the (Fab 8062)_3_/3-H complex was superimposed on a corresponding Fab of (Fab 8066)_3_/3-H complex using the Cα traces of the β-sheet of the variable domains (superimposed Fabs shown in purple), then (Fab 8062)_3_/3-H complexes were created using crystallographic symmetry operations of the Fab 8062 complex unit cell (shown in blue), or crystallographic symmetry operations of the Fab 8066 complex unit cell (shown in red). (B) (Fab 8062)_3_/3-H complex created using the crystallographic symmetry operations of the Fab 8066 complex unit cell shows multiple clashes between the side chains of the Fab 8062 (red) and 3-H (gray).

To verify that the requirement for Fabs 8066 and 8062 to adopt a different orientation upon binding to gp41 mimetics represents intrinsic properties of these antibodies, we carried out two modeling experiments. First, by superposing the respective β-sheet frameworks we have oriented a single Fab 8062 molecule (HL) into the orientation of the corresponding molecule of Fab 8066 in the complex with 3-H. Next, we applied the crystallographic symmetry operations of the Fab 8062 complex unit cell to the coordinates of Fab 8062 molecule rotated as above. This operation resulted in the creation of the complex that had very loose packing, with almost no interactions between the Fab molecules (Fig. 9A). In the second model the complex was created by applying the unit cell symmetry operators of Fab 8066 to the rotated molecule of Fab 8062. The resulting trimer revealed substantial clashes between the Fab molecules, as well as between the individual antibodies and the helices of 3-H (Fig. 9B).

Results of the modeling described above suggest that the changes in the orientation of Fabs 8066 and 8062 upon binding to 3-H are needed to create complexes in which packing of the individual components is optimal. Thus, variations in the sequences of CDRs H2 in two Fabs create obligatory adjustments in the structures of the complexes with gp41 mimetic (see below).

### Comparison of the Corresponding CDRs in Two 3-H Complexes

**Figure 10:**
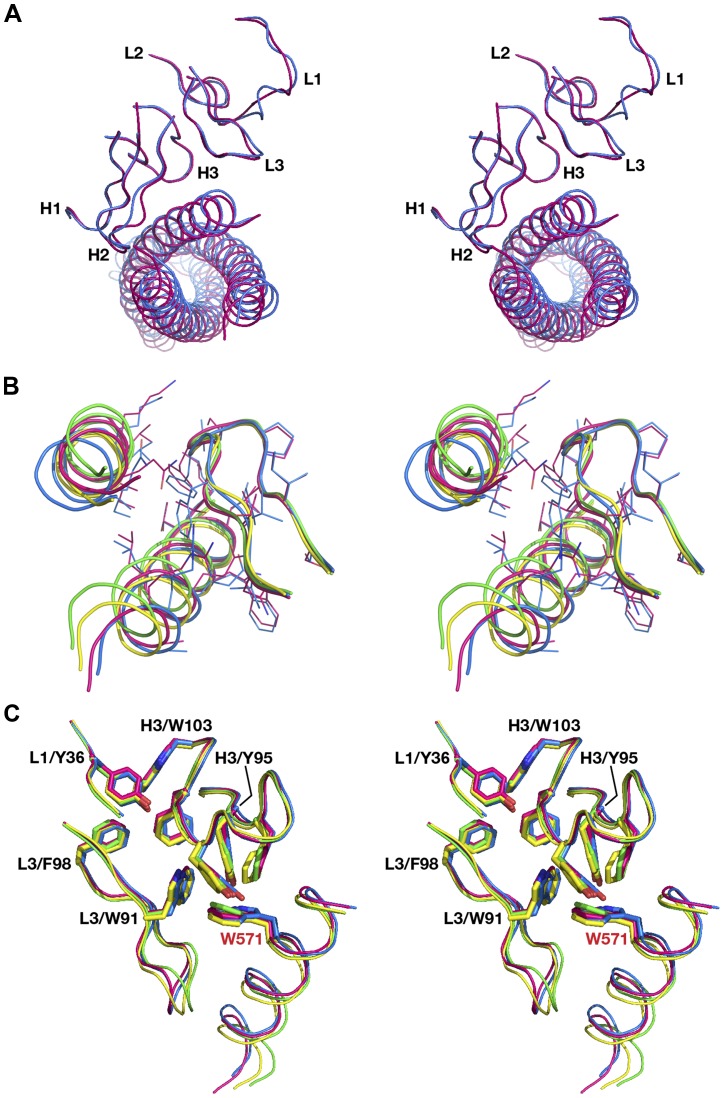
Comparison of the antigen-antibody interactions of single Fabs 8066 and 8062 in the complexes with 3-H and 5-Helix. Fabs were superimposed using the Cα traces of the β-sheet framework of the variable domains. CDRs and N-HR helices of the complexes of Fab 8066 with 3-H and 5-Helix are shown in red and green, respectively, while the corresponding fragments of the complexes of Fab 8062 are blue and yellow. (A) Superposition of all CDRs in two complexes with 3-H. (B) Contacts between CDRs H2 and two 2 N-HR helices in the four complexes. (C) A similar conformation of CDR H3 in the four complexes is stabilized by a cluster of aromatic residues from CDRs H3, L1 and L3. Residues F96, Y100B, and F100D of CDR H3 are displayed, but, for clarity, are not labeled.

Although the presence of three bound Fabs influences the structure of the 3-H molecule significantly, the interactions within the N-HR helix/Fab interface are mostly preserved (Table S2). The differences in the conformations of corresponding CDRs in two complexes can be easily seen in Fig. 10A, where two antibodies are oriented identically, by superimposing the β-sheet frameworks of their variable domains. Overall, the CDRs H2, L1 and L3 adopt different conformations in the two complexes, while the conformations of the CDRs H1, H3 and L2 are quite similar. CDR H2 has the longest interaction interface with CCIZN36 comprising residues L565–R579 of helix A plus residues V570, I573, K574, Q577 and L581 of helix B of 3-H (helix Nc in the 5-Helix complex). CDR H2 is the only one out of the six variable loops that makes contacts with two N-HR helices, whereas the CDRs H1, H3, L1, and L3 interact with only one N-HR helix (Fig. 10A,B). When this observation is combined with the fact that CDRs H2 are the only CDRs with a different sequence in the two antibodies, it seems reasonable to suggest, that the interactions of these particular CDRs with CCIZN36 have the most profound effect on the structure of 3-H compared to the others. Comparison of the interactions of the CDR H2 residues in the two complexes reveals contacts between four pairs of residues (F54/L565, T56/I573, T57/Q575, N58/W571), that are unique to the Fab 8066 complex, as opposed to two interacting pairs of residues (F56/I580, Q64/R579) unique to the Fab 8062 complex (Table S2).

CDRs H1 and H3 have very similar conformations in the two complexes interacting with the same set of the CCIZN36 residues in both complexes with a few more contacts in the Fab 8066 complex (Table S2). They interact with a similar segment of helix A, approaching the latter from two different sides. CDR H1 interacts with the two residues near the middle of the Fab/CCIZN36 interface (H564 and L568 in helix A). Similarly, CDR H3 makes contacts with residues Q563–W571 of the same helix. It is also of interest that the conformation of CDR H3 is supported by multiple interactions within a cluster of aromatic residues (Fig. 10C), composed of residues from three CDRs: Y95, F96, Y100B, F100D of CDR H3, Y36 of CDR L1, and W91 and F98 of CDR L3.

Two out of three CDRs of the light chains of the Fabs interact with CCIZN36. CDRs L2 do not interact with the antigen in either complex, and hence their conformations are quite similar (Fig. 10A). The conformations of the CDRs L1 and L3 vary in the two complexes due to differences in the interactions of the residues comprising them (Fig. 10A). In both complexes CDRs L1 are involved in interactions with the 71–74 loop of a neighboring antibody, although the number of contacts in the Fab 8062 complex is significantly larger (Table S2). CDR L1 also interacts in a similar way in both complexes with the residues Trp571 and Lys574 of helix A, but it makes additional contacts with the C-HR helix in the complexes with 5-Helix. CDR L3 interacts with a neighboring antibody only in the Fab 8066 complex. CDR L3 has a relatively short interaction interface with CCIZN36 towards the C-terminal end of the epitope, between Trp571 and Ala578 of helix A, with a similar number of contacts in the two complexes. In addition, in the complexes with 5-Helix two residues of CDR L3, Ser93 and Met94, interact with residues of the C-HR helix.

### Comparison of the Structures of N-HR Trimers in Two Complexes

**Figure 11:**
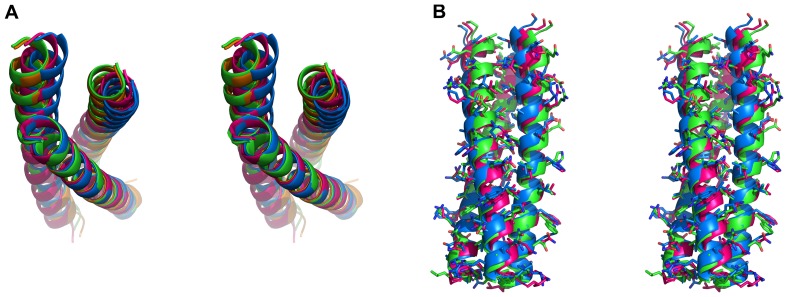
Superposition of the N-HR helices of gp41 in the (Fab)_3_/3-H complexes and in 6-helix bundles based on the Cα atoms of a single helix. (A) N-HR helices of the (Fab 8066)_3_/3-H complex (red), of the (Fab 8062)_3_/3-H complex (blue), of the 6-helix bundle of gp41 with a GCN4 zipper [25]: PDB code 1ENV (green), and of the 6-helix bundle of gp41 [24]; PDB code 1AIK (orange). (B) Shifts in the positions of the side chains in the N-HR helices of the 3 Fab 8062/3-Hcomplex (blue) with respect to their counterparts in the (Fab 8066)_3_/3-H complex (red) and 6-helix bundle (1ENV, green) indicates a greater degree of twist around the 3-fold axis of symmetry of the trimer.

As described above, comparison of the two complexes of Fabs 8066 and 8062 with 3-H reveals the differences in the mutual orientations of an antibody and the long axis of the N-HR trimer (Fig. 8B). In addition, binding of three Fabs to the exposed N-HR trimer causes some intrinsic perturbations of its structure. For comparisons of the trimers of N-HR helices we have also used, in addition to the structures presented here, the structures of the same antibodies complexed to 5-Helix (3MA9 and 3MAC), as well as the crystal structure of the six-helix bundle (6-HB) of gp41 ([24]; PDB code 1AIK). The 3-H construct includes an extra isoleucine zipper (IZ) which was added at the N terminus of the N36 sequence of gp41 to promote formation of a helical trimer [14]. To account for any potential impact of IZ on the structure of NHR trimer we also included in our comparisons the crystal structure of the 6-HB with a similar GCN4 sequence added to the N terminus ([25]; PDB code 1ENV). An overall comparison of the gp41 structures clearly indicates that there are small but significant differences between the exposed N-HR trimer structures and those in which C-HR helices are present (6-HB or 5-Helix). It is apparent from the comparison of the 6-HB structure, which includes a GCN4 zipper (1ENV) to the 6-HB that does not have it (1AIK) or to 5-Helix, that these differences cannot be attributed to the effect of the IZ zipper (Fig. 11A).

However, when the Fab 8062/3-H complex is compared to the Fab 8066/3-H complex, the N-HR trimer in the former complex exhibits a greater degree of twist around the 3-fold symmetry axis of the trimer (Fig. 11A). As a result, the side chains of the N-HR helices in the Fab 8062/3-H complex are much more displaced from their positions in the 6-Helix bundle, when the N-HR trimer structure from 6-HB (1ENV) is used as a reference, than in Fab 8066/3-H complex (Fig. 11B). This effect is particularly well illustrated when we use a morphing procedure to show the perturbations in the structure of N-HR trimers in motion (Movie S1). In this movie, we are using the structure of N-HR trimer from 6-HB as a starting point, and the coordinates of the corresponding trimers in the complexes with Fabs 8066 and 8062 as a reference point and a destination point, respectively. These results are in good agreement with the modeling conclusions from our previous study [2], which indicated that a more significant perturbation of the N-HR trimer was required to form a complex between three Fabs 8062 and 3-H.

### Comparison with 5-Helix (Both gp41 and Fabs)

Some data on comparison of the corresponding Fab complexes with 5-Helix and 3-H were discussed above. For example, it was shown that alignment of the four complexes based on the superposition of three N-HR helices demonstrates that the orientation of individual Fab molecules towards the long helix of a trimer varies in all of them (Fig. 8C). However, structural differences of the C termini of the helices Na and Nb in 5-Helix and 3-H are partially due to the different internal structure of the gp41 constructs. In 5-Helix, the C termini of helices Na and Nb are covalently linked to the C-helices that follow them, whereas in the CCIZN36 they are free.

**Figure 12:**
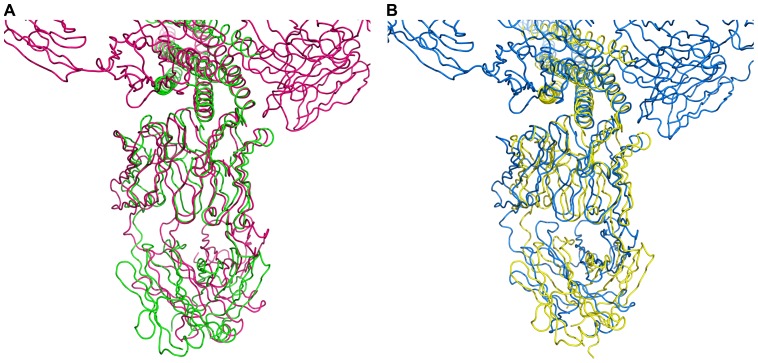
Superposition of the corresponding Fab 8066 and 8062 complexes with 3-H and 5-Helix based on the Cα coordinates of three N-HR helices. (A) The (Fab 8066)_3_/3-H (red) and the Fab 8066/5-Helix (green) complexes. (B) The (Fab 8062)_3_/3-H (blue) and Fab 8062/5-Helix (yellow) complexes.

It seems reasonable for clarity to separate the two sources of the differences in the structures of the four complexes that are being compared. The differences in the binding of two antibodies to the same target (either 3-H or 5-Helix) originate from the differences in the sequences of their CDRs H2. If we compare the binding of the corresponding Fabs to two different gp41 mimetics (3-H and 5-Helix) separately, we also notice, that the Fab β-sheet framework in the Fab/3-H trimer complexes is positioned differently in relation to the 3-fold axis of symmetry of the trimer compared to the Fab/5-Helix complexes (Fig. 12). These differences are consistently more significant in the Fab 8062/3-H/5-Helix complexes then in the Fab 8066/3-H/5-Helix complexes (Figs. 8C and 12). In the complexes with 3-H the displacement of the β-sheet framework appears to originate from the close contacts between the antibodies. They include the interactions between the L1 variable loop and the loop from the neighboring Fab in the two 3 Fab/3-H complexes, involving residues around Ser74 (loop 74, Table S3). This observation is reminiscent of affinity maturation effects observed previously [23], [26]. It has been noted in previous studies that, besides the gain in favorable interactions, affinity maturation also results in resolving unfavorable contacts, such as the steric limitations we observed here. In the complexes with 5-Helix a similar role is played by the C-HR helices. The contacts of C-helices with Fabs 8066 and 8062 tune the orientation of each antibody towards 5-Helix.

**Figure 13:**
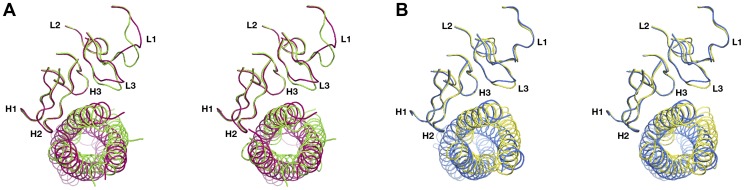
Pairwise comparison of all CDRs in a single antibody. The complexes of Fabs 8066 (A) and 8062 (B) with 3-H and 5-Helix were superimposed using the Cα traces of the β-sheet frameworks of the variable domains. N-HR helices and CDR loops of the (Fab 8066)_3_/3-H complex are shown in red, of the 8066/5-Helix complex are in green, of the (Fab 8062)_3_/3-H complex are in blue, and of the 8062/5-Helix complex are in yellow.

Superposition of the complexes based on the β-sheet framework of the antibody demonstrates the differences in the structure of N-HR trimers in all four complexes (Fig. 8D) described in detail in the previous paragraph. In addition, such superposition yields very interesting results for the comparison of the conformations of the respective CDRs in the complexes of each antibody with 3-H and 5-Helix. As can be seen in Fig. 13, when two complexes of each antibody, bound to either 3-H or 5-Helix, are superimposed using their β-sheet framework as a basis, it results in nearly perfect alignment of all CDRs, with the exception of the CDRs of the light chain, which are involved either in interactions with neighboring antibodies in the complexes with 3-H, or/and in interactions with C-HR helices in the complexes with 5-Helix (Tables S2, S3). Such contacts between the neighboring antibodies (or between the antibody and C-HR helices in 5-Helix) serve as an additional source of conformational changes displacing the β-sheet framework of the antibodies.

## Discussion

The profound differences in the biological activities of the Fabs 8066 and 8062 were difficult to explain by comparing the structures of their complexes with 5-Helix. It has been previously shown that deactivation of gp41 in vivo is a slow, reversible process that is dependent on chemokine receptor binding to Env, and that the exposed N-HR trimer remains accessible to inhibitors until the final conformational changes in gp41 that lead to formation of the 6-HB have taken place [27]. This observation suggests that binding of more than one antibody to the N-HR trimer may potentiate inhibition of fusion. Our previous modeling efforts which were based on the structures of the complexes of the two Fabs with 5-Helix led to conclusions that the binding of three molecules of Fab 8066 to a N-HR trimer could be accomplished without significant steric clashes, whereas the binding of multiple Fab 8062 molecules seemed more problematic. We have chosen a different gp41 mimetic ((CCIZN36)_3_, here called 3-H), which presents the exposed N-terminal trimeric coiled coil, in order to obtain experimental structural and biophysical data on the complexes of gp41 with multiple antibodies. Binding studies of the complexes between 3-H and Fabs 8066 and 8062 were complicated by the very high affinity of the interactions (K_d_<10 nM), as well as saturation and statistical effects arising from sequential binding of 3 Fabs to one target molecule. Appearance of the intermediate populated species (3-H trimer with one and two Fabs bound) during the Fab titration monitored by analytical ultracentrifugation allowed us to deduce that the three binding sites are independent (no significant positive or negative cooperativity effects). The direct observation of molecular images of 3-H complexes with different stoichiometries by cryo-electron microscopy can also be readily interpreted using the crystallographic structure (Fig. S5 in File SI). Global analysis of the complex dissociation isotherms [16], monitored by intrinsic tryptophan fluorescence, sedimentation equilibrium experiments, and ITC titrations yielded K_d_ values for Fab 8066 and Fab 8062 interactions with 3-H that are essentially indistinguishable (4.3 and 4.2 nM, respectively), which is significantly different from the previously determined K_d_s for Fab 8066 and Fab 8062 interactions with 5-Helix (<10 nM and 200 nM , respectively [2]).

A detailed, comparative analysis of the structures of two complexes with 3-H revealed fine, but important differences in the mutual orientation of the antibody molecule and the trimer of N-HR helices, as well as the differences in the structure of the trimer itself. As described above, attempts to recreate the complex of three Fab 8062 with 3-H from a model, containing one N-HR helix and a single Fab 8062 molecule with a relative orientation of Fab 8066 bound to 3-H, applying crystallographic symmetry operations of either complexes, were not successful (Fig. 9). This result indicates that differences in the structures of two complexes are essential and required for the tight fit of CDR H2 of Fab 8062. These adjustments in the orientation of the individual antibody molecules upon binding to 3-H reflect intrinsic properties of the two Fabs. They directly correlate with changes in the structure of the antigen and can be achieved as a consequence of the flexible nature of 3-H as opposed to 5-Helix, that is more rigid due to the presence of the C-HR helices. These data explain well the differences in the binding constants of Fab 8062 to 5-Helix vs. 3-H. Pairwise comparison of the structures of the two antibodies bound to the same antigen shows that consistently larger rearrangements are required for the successful complex formation of Fab 8062.

The lack of neutralization activity for Fab 8062 indicates that an optimized fit of the antibody and the required changes in the structure of the antigen cannot be achieved (or sustained) for the 3 Fab 8062/3-H complex formation in the context of viral entry. One can propose a number of potential explanations for this finding. Most likely as part of the gp41 protein in the context of the functional envelope, a trimer of N-HR helices is subjected to significant conformational constraints. The kinetics of gp120 dissociation may potentially also play a role, as a recent cryo-EM study has shown that in the cross-linked gp140 structure, the gp41 pre-hairpin intermediate exhibits a much more expanded N-HR trimer structure than that previously observed in the 6-HB and in this study [28]. It is also possible that the actual formation of 6-HB restricts the conformational space available to the pre-hairpin intermediate of gp41. It is evident from the structural data presented here that binding of C-HR helices prohibits changes in N-HR trimer structure, and given the fact that the exact timing of the events that comprise the HIV entry process is not known, it is conceivable that C-HR helices may bind in sequential manner, thus preventing the N-HR trimer structure from changing [27]. In this case some antibodies that were generated against N-HR trimer, such as Fab 8062, which adopted the binding mode with high affinity due to the induced conformational changes in the antigen, are at a disadvantage. This conclusion is in agreement with the findings by Zwick and colleagues [29] which showed that a combination of gp41 mimetics was required to isolate neutralizing antibodies against gp41. Therefore, our structural results indicate that high affinity of an antibody towards both gp41 mimetics that contain C-helices and to the exposed N-HR trimer might be the key feature defining this antibody as potentially biologically active molecule.

A comparison of the complexes consisting of (Fabs 8062)_3_/3-H and (Fab 8066)_3_/3-H indicates that the extensive interaction interface of CDR-H2 dominates complex formation to the point that the architecture of the whole complex is changed to accommodate differences in the CDR-H2 conformation. The 3-H is flexible enough to accommodate such a change, which results in very high affinity binding of Fab 8062. However the fact that Fab 8062 shows no neutralizing activity clearly demonstrates that, in the context of an intact virus, the pre hairpin-intermediate cannot adapt to Fab 8062 as easily as to Fab 8066, making this mode of binding not biologically relevant. Thus Fab 8062 represents a dead-end of the antibody evolution. Using a more rigid target antigen for CDR-H2 optimization that does not allow such significant structure perturbations to take place (such as 5-Helix) would have eliminated Fab 8062 from the affinity optimized antibody pool. On the other hand, since 5-Helix allows for only one Fab binding, it eliminates the constraints imposed by the binding of the neighboring Fabs in the (Fab 8066)_3_/3-H complex on the conformation of CDR-H2, L3 and L1 conformations. Thus neither of these two antigen-mimetics adequately represents all the structural features of the pre-hairpin intermediate of gp41 and the combination of both may be required for successful selection and further optimization of neutralizing antibodies. Comparison of the binding affinities of an antibody towards antigen-mimetics of gp41 that contain an NH trimer in the absence or presence of one or two C-helices would show whether such antibody retains its high affinity during the intermediate stages of the six-helix bundle formation. As unambiguously shown in this study, such data would provide a good criterion for the selection of potentially neutralizing antibodies.

The importance of structural information in immunogen design is further supported by a growing body of evidence that extensive selection procedures are required for isolation of biologically relevant active fraction of antibodies generated either by natural selection in the infected individual [30] or in the immunization efforts [31].

## Materials and Methods

### Protein Expression and Purification

The gene sequence encoding the CCIZN36 peptide (corresponding to residues 546–582 of HIV-1 gp41 [14]) was subcloned into a modified pET-32a vector [32] to form a thioredoxin fusion protein with a His_6_ tag, and was expressed in the *Escherichia coli* strain BL21(DE3) (Novagen, La Jolla, CA). The construct was verified by DNA sequencing and the molecular mass of the expressed fusion protein (24779.5±2.1 Da) was verified by electrospray mass spectrometry. *E. coli* transformed with the CCIZN36 vector was grown in Luria Bertani medium at 37°C, induced with 1 mM isopropyl D-thiogalactopyranoside at A600 = 0.8, and harvested by centrifugation 3 h following induction. After harvesting, the cell pellet was resuspended in 50 ml (per liter of culture) of 25 mM Tris, pH 7.4, 6 M guanidinium HCl and 10 mM imidazole; the suspension was lysed by sonication and centrifuged at 16,000 g for 20 min. The supernatant fraction was loaded onto a Ni-NTA column (5 ml; QIAGEN, Germantown, MD) washed with 25 mM Tris, pH 7.4, 1 M NaCl, and 25 mM Tris, pH 7.4, 200 mM NaCl, and the fusion protein was digested with thrombin (10 NIH units/mg of protein). The CCIZN36 peptide was eluted with pH gradient from the starting buffer to 20 mM sodium acetate pH 5.2, 200 mM NaCl, and further purified by reverse-phase HPLC using PROTO C4 semi-preparative column (Higgins Analytical, Inc., Mountain View, CA) and size exclusion chromatography using Superdex-75 gel filtration column (GE Healthcare, Waukesha, WI) equilibrated with 20 mM sodium acetate pH 5.2, 200 mM NaCl. The molecular mass of the purified 3-H (22674.8±22.8) was verified using MALDI-TOF mass spectrometry (Fig. S1 in File SI).

Recombinant mini-antibodies were obtained as described previously [7]. Antibodies were further purified by size exclusion chromatography using a Superdex-200 column (GE Healthcare, Waukesha, WI) eluting with 20 mM sodium acetate pH 5.2, 200 mM NaCl, and peak fractions were pooled and concentrated. The masses of the heavy (26281.7±3.5 Da for 8062, and 26278.0±3.5 Da for 8066) and the light (22624.2±3.3 Da) chains were verified by electrospray mass spectrometry.

3 Fab/3-H complexes for crystallization trials were produced by mixing dilute purified 3-H with purified Fabs, followed by fractionation on Superdex-200 column (GE Healthcare, Waukesha, WI) in 20 mM sodium acetate, pH 5.2, 200 mM NaCl, peak fractions corresponding to the complex were pooled and concentrated.

### Sedimentation Velocity

Sedimentation velocity experiments were carried out at 25.0°C and 50,000 rpm on a Beckman Coulter ProteomeLab XL-I analytical ultracentrifuge following standard protocols [33]. Absorbance (230 or 280 nm) and Rayleigh interference (655 nm) scans were collected at approximately 7 minute intervals and data were analyzed in SEDFIT 14.1 [34] in terms of a continuous *c(s)* distribution with a resolution of 0.05 S and a confidence level 0.68. Scan file time-stamps were corrected [35] and good fits were obtained with r.m.s.d. values corresponding to typical instrument noise values. The solution density (ρ) and viscosity (η) were determined experimentally at 20.000°C on an Anton-Paar DMA 5000 density meter and 20.00°C using an Anton Paar AMVn rolling ball viscometer, respectively and corrected to values at 25.0°C. Protein partial specific volumes were calculated in SEDNTERP 1.09 [36] based on the amino acid composition.

All samples were prepared in 10 mM MES, 150 mM NaCl (pH 5.6) and studied at various loading concentrations. Sedimentation coefficients were corrected to standard conditions, *s_20,w_* and weighted average sedimentation coefficient *s_w_* isotherms were generated by integrating the *c(s)* distributions. Protein extinction coefficients at 280 nm and interference signal increments at 655 were calculated based on the amino acid composition in SEDFIT 14.1 [37].

### Determination of the Binding Affinities

Isothermal titration calorimetry data were collected using a VP-ITC titration calorimeter (MicroCal, GE Healthcare, Waukesha, WI.). All samples were prepared in 10 mM MES, 150 mM NaCl, pH 5.6. Raw data were baseline corrected and integrated in NITPIC 0.7.7 beta [38], and exported for analysis into SEDPHAT 10.55b [39]. Isothermal titration calorimetry data were plotted in GUSSI 1.0.4 U (https:/sedfitsedphat.nibib.nih.gov/software). All experiments were carried out at 25.0°C.

Fluorescence intensity data for dilution series of the purified complexes of (Fab)_3_/3-H complexes were collected with a FluoroMax-3 (HORIBA Scientific, Edison, NJ) instrument at 325 and 355 nm, using an excitation wavelength of 280 nm. Buffer conditions were the same as those utilized in the sedimentation velocity experiments (see above). Differences between the 325 nm and 355 nm data were calculated and used for analysis – studies on the individual components were used to determine fluorescent signal increments SI_325–355_ (Fig. S2 in File SI). Due to the negative value of SI_325–355_ for the 3-H, fluorescence data for the study of the interaction were corrected for contributions from the individual components resulting in isotherms that contain only signal contributions that arise from binding. Data were analyzed in SEDHAT 10.55b in terms of an A+B+B+B = AB + B + B = ABB + B = ABBB model with 3 symmetric sites and a macroscopic K. The analyses assumed the following: (i) no positive or negative cooperativity with log_10_(K_a2_/K_a1_) = −0.47710 and log_10_(K_a3_/K_a1_) = −0.95420 where K_a1_, K_a2_ and K_a3_ represent the macroscopic equilibrium constants for the binding of the first, second and third antibody; (ii) identical enthalpies describing antibody binding at all sites irrespective of occupancy with ΔH(AB)B – ΔH(AB) = ΔH(ABB)B - ΔH(AB)B = 0 where ΔH(AB), ΔH(AB)B and ΔH(ABB)B represent the reaction enthalpy for the binding of the first, second and third antibody and (iii) a complex macroscopic signal change SI_325–355_ per binding interface that is identical for each antibody binding event with SI_325–355_ ABB/SI_325–355_ AB = SI_325–355_ ABBB/SI_325–355_ AB = 1. As data were corrected for SI_325–355_ contributions from the 3-H and antibody and molar signal increments cannot be set to zero, these were set to a very small number (namely 1) for the purposes of data analysis (see Fig. S2 in File SI for actual molar signal increments).

### Procedures for Cryo-electron Microscopy

Fab 8066/3-H complexes purified by gel filtration were deposited on Quantifoil R22 grids, blotted at 100% humidity and plunge-frozen in an FEI Mark III Vitrobot into a container of liquid ethane cooled by liquid nitrogen using standard procedures for vitrification as described previously [28]. The frozen grids were imaged in a FEI Titan Krios electron microscope at an electron dose of ∼20 electrons/Å^2^, with the microscope operated at 80 kV. Projection images were recorded using a Falcon II detector.

### Crystallographic Procedures

Crystallization of the (Fab 8066)_3_/3-H and (Fab 8062)_3_/3-H complexes was carried out by the hanging drop, vapor diffusion method. The (Fab 8066)_3_/3-H complex was concentrated to 7 mg/ml in 20 mM sodium acetate buffer pH 5.2, also containing 0.2 M NaCl. The reservoir solution contained 15% PEG4000 with 0.2 M ammonium sulfate in 0.1 M sodium citrate buffer pH 5.5. Each drop contained 1 µl of protein and 1 µl of reservoir solution. The crystals grew to their maximum size of ∼0.2 mm in 4 to 5 days. The (Fab 8062)_3_/3-H complex was treated in a same way. Diffraction data for both complexes were collected using synchrotron radiation at the SER-CAT ID-22 beamline at the APS, Argonne National Laboratory, from one crystal each, and were processed with HKL2000 [40]. Data resolution for the (Fab 8066)_3_/3-H and (Fab 8062)_3_/3-H complexes was 2.8 and 3.0 Å, respectively (Table 1). The structure of the (Fab 8066)_3_/3-H complex was solved by molecular replacement with the program PHASER [41] in two steps. In the first step a partial search model was generated from the structure of the Fab 8066/5-Helix complex (PDB ID 3MA9). One inner N-HR helix from the 5-Helix bundle and the variable domains of the light and heavy chains were used to search the (Fab 8066)_3_/3-H diffraction data. In the second step, the constant domains of Fab 8066 were located using the corresponding domains from the Fab 8066/5-Helix complex as the search model. The structure was completed through a number of cycles of refinement with REFMAC5 [42] and rebuilding with COOT [20]. The refined structure of the (Fab 8066)_3_/3-H complex was used to generate the search models to solve the structure of the (Fab 8062)_3_/3-H complex in two steps, with similar procedures used for subsequent refinement and model building. The statistics for data collection and structure refinement are listed in Table 1. The coordinates and structure factors for the (Fab 8066)_3_/3-H and (Fab 8062)_3_/3-H complexes have been deposited in the Protein Data Bank with IDs 4KHT and 4KHX, respectively.

## Supporting Information

File SI **Five supporting figures and three supporting tables. Figure S1, SDS-PAGE of purified CCIZN36 disulfide-linked trimer under non-reducing and reducing conditions.** Lane1, low molecular weight standards (GE Healthcare, Waukesha, WI), Lane 2, purified 3-H under non-reducing conditions (molecular weight 22674.8±22.8 Da verified using MALDI-TOF mass spectrometry), Lane 3, purified 3-H under reducing conditions (molecular weight of the monomer 7559.8±2.5 Da and dimer 15123.6±39.0 Da verified using MALDI-TOF mass spectrometry). **Figure S2, Fluorescence signal increments.** 325 and 355 nm fluorescence emission intensity differences for (A) the 3-H and (B) the 8062 (blue) and 8066 (red) antibodies as a function of loading concentration. Fluorescence signal increments, SI325–355 of −2.52×10^11^ M^−1^, 2.68×10^10^ M^−1^ and 1.12×10^11^ M^−1^ are determined for the 3-H, 8062 and 8066 antibodies, respectively. **Figure S3, Crystal packing of the (Fab)3/3-H complexes.** (A) Complexes of Fab 8066 are aligned “head to tail”. Each asymmetric unit contains one Fab and one N-HR helix (shown in different colors). (B) Helices of 3-H trimers form an infinite helix in the crystal. Hydrogen bonds between different 3-H trimers are shown in black. **Figure S4, Superposition of the (Fab 8066)3/3-H complex (red) and (Fab 8062)3/3-H complex (blue).** The superposition was based on Cα atoms of a β-sheet framework of the variable domain of a single Fab. **Figure S5, Selected examples of a single projection molecular images.** The putative occupancies of Fab 8066 bound to the gp41 trimer are 1 (A), 2 (B), or 3 (C). Projection views of the crystallographically determined structure of the gp41-8066 complex are shown to mimic the orientation of the selected molecular images. The molecular structures shown in panels A and B were generated by removing either two copies or one copy, respectively of the 8066 Fab fragment, while the structure shown in panel C is that of the intact trimer with the bound Fab 8066. The orientations of the complexes were adjusted manually to show the best agreement with the electron microscopic images. **Table S1, Residue numbering of gp41 N-helices in 3 Fab/(CCIZN36)_3_ complexes, Fab/5-Helix complexes and native full-length gp41.** For the 5-Helix complex with Fab 8066 helices are highlighted with gray boxes and residues not visible in the electron density map are shown in small letters. For the 3 Fab/(CCIZN36)_3_ complexes the helices are continuous and all residues are visible. Table S2, Antigen-antibody interactions, all with helix A (Na in 5-Helix), except where indicated, helix B is Nc in 5-Helix, C helix residues in italics, Hydrophobic contacts in bold, *Hydrogen bonds/polar contacts. **Table S3, Antibody-antibody contacts in 3 8066/(CCIZN36)_3_ and 3 8062/(CCIZN36)_3_.** Hydrophobic contacts in bold, *Hydrogen bonds/polar contacts.
(PDF)

Movie S1 **Changes in the structure of the N-trimer induced by the antibody binding.** The orientation of the N-trimer in the crystal structure of a six-helix bundle of gp41, shown in yellow (PDB code 1ENV, C-helices are shown in white) is used as a starting point. The morphing procedure is applied to the N-trimer from the 6-HB to adopt the orientation of the N-trimer in the complex with Fab 8062 that is used as the destination point (green). The corresponding trimer in the complex with Fab 8066 (shown in red) is used as a reference point. The N-trimers from three structures are superimposed using the Cα coordinates of a single N helix. The side chains are added stepwise as sticks of the corresponding colors.
(MOV)

## References

[1] Luftig MA, Mattu M, Di Giovine P, Geleziunas R, Hrin R, et al (2006) Structural basis for HIV-1 neutralization by a gp41 fusion intermediate-directed antibody. Nat Struct Mol Biol 13: 740–747. 16862157 10.1038/nsmb1127

[2] Gustchina E, Li M, Louis JM, Anderson DE, Lloyd J, et al (2010) Structural basis of HIV-1 neutralization by affinity matured Fabs directed against the internal trimeric coiled-coil of gp41. PLoS Pathog 6: e1001182. 21085615 10.1371/journal.ppat.1001182 PMC2978731

[3] Sabin C, Corti D, Buzon V, Seaman MS, Lutje HD, et al (2010) Crystal structure and size-dependent neutralization properties of HK20, a human monoclonal antibody binding to the highly conserved heptad repeat 1 of gp41. PLoS Pathog 6: e1001195. 21124990 10.1371/journal.ppat.1001195 PMC2987821

[4] Merk A, Subramaniam S (2013) HIV-1 envelope glycoprotein structure. Curr Opin Struct Biol 23: 268–276. 23602427 10.1016/j.sbi.2013.03.007 PMC3676719

[5] Harris AK, Bartesaghi A, Milne JL, Subramaniam S (2013) HIV-1 Envelope Glycoprotein Trimers Display Open Quaternary Conformation When Bound to the gp41 Membrane-Proximal External-Region-Directed Broadly Neutralizing Antibody Z13e1. J Virol 87: 7191–7196. 23596305 10.1128/JVI.03284-12 PMC3676106

[6] Gustchina E, Louis JM, Lam SN, Bewley CA, Clore GM (2007) A monoclonal Fab derived from a human nonimmune phage library reveals a new epitope on gp41 and neutralizes diverse human immunodeficiency virus type 1 strains. J Virol 81: 12946–12953. 17898046 10.1128/JVI.01260-07 PMC2169134

[7] Gustchina E, Louis JM, Frisch C, Ylera F, Lechner A, et al (2009) Affinity maturation by targeted diversification of the CDR-H2 loop of a monoclonal Fab derived from a synthetic naive human antibody library and directed against the internal trimeric coiled-coil of gp41 yields a set of Fabs with improved HIV-1 neutralization potency and breadth. Virol 393: 112–119. 10.1016/j.virol.2009.07.019 PMC2759307 19695655

[8] Rothe C, Urlinger S, Lohning C, Prassler J, Stark Y, et al (2008) The human combinatorial antibody library HuCAL GOLD combines diversification of all six CDRs according to the natural immune system with a novel display method for efficient selection of high-affinity antibodies. J Mol Biol 376: 1182–1200. 18191144 10.1016/j.jmb.2007.12.018

[9] Louis JM, Bewley CA, Clore GM (2001) Design and properties of N(CCG)-gp41, a chimeric gp41 molecule with nanomolar HIV fusion inhibitory activity. J Biol Chem 276: 29485–29489. 11418583 10.1074/jbc.C100317200

[10] Krey T, Meola A, Keck ZY, Damier-Piolle L, Foung SK, et al (2013) Structural basis of HCV neutralization by human monoclonal antibodies resistant to viral neutralization escape. PLoS Pathog 9: e1003364. 23696737 10.1371/journal.ppat.1003364 PMC3656090

[11] Root MJ, Kay MS, Kim PS (2001) Protein design of an HIV-1 entry inhibitor. Science 291: 884–888. 11229405 10.1126/science.1057453

[12] Bianchi E, Finotto M, Ingallinella P, Hrin R, Carella AV, et al (2005) Covalent stabilization of coiled coils of the HIV gp41 N region yields extremely potent and broad inhibitors of viral infection. Proc Natl Acad Sci U S A 102: 12903–12908. 16129831 10.1073/pnas.0502449102 PMC1200264

[13] Bianchi E, Joyce JG, Miller MD, Finnefrock AC, Liang X, et al (2010) Vaccination with peptide mimetics of the gp41 prehairpin fusion intermediate yields neutralizing antisera against HIV-1 isolates. Proc Natl Acad Sci U S A 107: 10655–10660. 20483992 10.1073/pnas.1004261107 PMC2890830

[14] Eckert DM, Kim PS (2001) Design of potent inhibitors of HIV-1 entry from the gp41 N-peptide region. Proc Natl Acad Sci U S A 98: 11187–11192. 11572974 10.1073/pnas.201392898 PMC58705

[15] Cai M, Huang Y, Suh JY, Louis JM, Ghirlando R, et al (2007) Solution NMR structure of the barrier-to-autointegration factor-Emerin complex. J Biol Chem 282: 14525–14535. 17355960 10.1074/jbc.M700576200

[16] Zhao H, Schuck P (2012) Global multi-method analysis of affinities and cooperativity in complex systems of macromolecular interactions. Anal Chem 84: 9513–9519. 23020071 10.1021/ac302357w PMC3491091

[17] Karle IL, Gopi HN, Balaram P (2003) Crystal structure of a hydrophobic 19-residue peptide helix containing three centrally located D amino acids. Proc Natl Acad Sci U S A 100: 13946–13951. 14617766 10.1073/pnas.2336106100 PMC283526

[18] Cohen GE (1997) ALIGN: a program to superimpose protein coordinates, accounting for insertions and deletions. J Appl Crystallogr 30: 1160–1161.

[19] Krissinel E, Henrick K (2004) Secondary-structure matching (SSM), a new tool for fast protein structure alignment in three dimensions. Acta Crystallogr D60: 2256–2268. 10.1107/S0907444904026460 15572779

[20] Emsley P, Cowtan K (2004) Coot: model-building tools for molecular graphics. Acta Crystallogr D60: 2126–2132. 10.1107/S0907444904019158 15572765

[21] Stanfield RL, Zemla A, Wilson IA, Rupp B (2006) Antibody elbow angles are influenced by their light chain class. J Mol Biol 357: 1566–1574. 16497332 10.1016/j.jmb.2006.01.023

[22] DeLano WL (2002) The PyMOL Molecular Graphics System. San Carlos, CA: DeLano Scientific.

[23] Bajorath J, Harris L, Novotny J (1995) Conformational similarity and systematic displacement of complementarity determining region loops in high resolution antibody x-ray structures. J Biol Chem 270: 22081–22084. 7673180 10.1074/jbc.270.38.22081

[24] Chan DC, Fass D, Berger JM, Kim PS (1997) Core structure of gp41 from the HIV envelope glycoprotein. Cell 89: 263–273. 9108481 10.1016/s0092-8674(00)80205-6

[25] Weissenhorn W, Dessen A, Harrison SC, Skehel JJ, Wiley DC (1997) Atomic structure of the ectodomain from HIV-1 gp41. Nature 387: 426–430. 9163431 10.1038/387426a0

[26] Krause JC, Ekiert DC, Tumpey TM, Smith PB, Wilson IA, et al (2011) An insertion mutation that distorts antibody binding site architecture enhances function of a human antibody. MBio 2: e00345–10. 21304166 10.1128/mBio.00345-10 PMC3037006

[27] Kahle KM, Steger HK, Root MJ (2009) Asymmetric deactivation of HIV-1 gp41 following fusion inhibitor binding. PLoS Pathog 5: e1000674. 19956769 10.1371/journal.ppat.1000674 PMC2776349

[28] Tran EE, Borgnia MJ, Kuybeda O, Schauder DM, Bartesaghi A, et al (2012) Structural mechanism of trimeric HIV-1 envelope glycoprotein activation. PLoS Pathog 8: e1002797. 22807678 10.1371/journal.ppat.1002797 PMC3395603

[29] Nelson JD, Nelson JD, Kinkead H, Brunel FM, Leaman DJ, et al (2008) Antibody elicited against the gp41 N-heptad repeat (NHR) coiled-coil can neutralize HIV-1 with modest potency but non-neutralizing antibodies also bind to NHR mimetic. Virology 377: 170–183. 18499210 10.1016/j.virol.2008.04.005 PMC2493441

[30] Walker LM, Simek MD, Priddy F, Gach JS, Wagner D, et al (2010) A limited number of antibody specificities mediate broad and potent serum neutralization in selected HIV-1 infected individuals. PLoS Pathog 6: e10001028. 10.1371/journal.ppat.1001028 PMC2916884 20700449

[31] Cheung WC, Beausoleil SA, Zhang X, Sato S, Schieferl SM, et al (2012) A proteomics approach for the identification and cloning of monoclonal antibodies from serum. Nat Biotechnol 30: 447–452. 22446692 10.1038/nbt.2167

[32] Cai M, Williams DC Jr, Wang G, Lee BR, Peterkofsky A, et al (2003) Solution structure of the phosphoryl transfer complex between the signal-transducing protein IIAGlucose and the cytoplasmic domain of the glucose transporter IICBGlucose of the Escherichia coli glucose phosphotransferase system. J Biol Chem 278: 25191–25206. 12716891 10.1074/jbc.M302677200

[33] Zhao H, Brautigam CA, Ghirlando R, Schuck P (2013) Overview of current methods in sedimentation velocity and sedimentation equilibrium analytical ultracentrifugation. Curr Protoc Protein Sci Chapter 20: Unit20. 10.1002/0471140864.ps2012s71 PMC3652391 23377850

[34] Schuck P (2000) Size-distribution analysis of macromolecules by sedimentation velocity ultracentrifugation and lamm equation modeling. Biophys J 78: 1606–1619. 10692345 10.1016/S0006-3495(00)76713-0 PMC1300758

[35] Zhao H, Ghirlando R, Piszczek G, Curth U, Brautigam CA, et al (2013) Recorded scan times can limit the accuracy of sedimentation coefficients in analytical ultracentrifugation. Anal Biochem 437: 104–108. 23458356 10.1016/j.ab.2013.02.011 PMC3676908

[36] Cole JL, Lary JW, Moody P, Laue TM (2008) Analytical ultracentrifugation: sedimentation velocity and sedimentation equilibrium. Methods Cell Biol 84: 143–179. 17964931 10.1016/S0091-679X(07)84006-4 PMC2711687

[37] Zhao H, Brown PH, Schuck P (2011) On the distribution of protein refractive index increments. Biophys J 100: 2309–2317. 21539801 10.1016/j.bpj.2011.03.004 PMC3149238

[38] Keller S, Vargas C, Zhao H, Piszczek G, Brautigam CA, et al (2012) High-precision isothermal titration calorimetry with automated peak-shape analysis. Anal Chem 84: 5066–5073. 22530732 10.1021/ac3007522 PMC3389189

[39] Schuck P (2003) On the analysis of protein self-association by sedimentation velocity analytical ultracentrifugation. Anal Biochem 320: 104–124. 12895474 10.1016/s0003-2697(03)00289-6

[40] Otwinowski Z, Minor W (1997) Processing of X-ray diffraction data collected in oscillation mode. Methods Enzymol 276: 307–326. 10.1016/S0076-6879(97)76066-X 27754618

[41] McCoy AJ, Grosse-Kunstleve RW, Adams PD, Winn MD, Storoni LC, et al (2007) *Phaser* crystallograhic software. J Appl Cryst 40: 658–674. 19461840 10.1107/S0021889807021206 PMC2483472

[42] Murshudov GN, Skubak P, Lebedev AA, Pannu NS, Steiner RA, et al (2011) REFMAC5 for the refinement of macromolecular crystal structures. Acta Crystallogr D67: 355–367. 10.1107/S0907444911001314 PMC3069751 21460454

[43] Brünger AT (1992) The free R value: a novel statistical quantity for assessing the accuracy of crystal structures. Nature 355: 472–474. 18481394 10.1038/355472a0

