# Supplementary material for: Complexes of Neutralizing and Non-Neutralizing Affinity Matured Fabs with a Mimetic of the Internal Trimeric Coiled-Coil of HIV-1 gp41
Source: PLoS One. 2013 Nov 7;8(11):e78187. doi: 10.1371/journal.pone.0078187 (PMC3820714; doi:10.1371/journal.pone.0078187)
Supplement: File SI — Five supporting figures and three supporting tables. Figure S1, SDS-PAGE of purified CCIZN36 disulfide-linked trimer under non-reducing and reducing conditions. Lane1, low molecular weight standards (GE Healthcare, Waukesha, WI), Lane 2, purified 3-H under non-reducing conditions (molecular weight 22674.8±22.8 Da verified using MALDI-TOF mass spectrometry), Lane 3, purified 3-H under reducing conditions (molecular weight of the monomer 7559.8±2.5 Da and dimer 15123.6±39.0 Da verified using MALDI-TOF mass spectrometry). Figure S2, Fluorescence signal increments. 325 and 355 nm fluorescence emission intensity differences for (A) the 3-H and (B) the 8062 (blue) and 8066 (red) antibodies as a function of loading concentration. Fluorescence signal increments, SI325–355 of −2.52×10^11^ M−1, 2.68×10^10^ M−1 and 1.12×10^11^ M−1 are determined for the 3-H, 8062 and 8066 antibodies, respectively. Figure S3, Crystal packing of the (Fab)3/3-H complexes. (A) Complexes of Fab 8066 are aligned “head to tail”. Each asymmetric unit contains one Fab and one N-HR helix (shown in different colors). (B) Helices of 3-H trimers form an infinite helix in the crystal. Hydrogen bonds between different 3-H trimers are shown in black. Figure S4, Superposition of the (Fab 8066)3/3-H complex (red) and (Fab 8062)3/3-H complex (blue). The superposition was based on Cα atoms of a β-sheet framework of the variable domain of a single Fab. Figure S5, Selected examples of a single projection molecular images. The putative occupancies of Fab 8066 bound to the gp41 trimer are 1 (A), 2 (B), or 3 (C). Projection views of the crystallographically determined structure of the gp41-8066 complex are shown to mimic the orientation of the selected molecular images. The molecular structures shown in panels A and B were generated by removing either two copies or one copy, respectively of the 8066 Fab fragment, while the structure shown in panel C is that of the intact trimer with the bound Fab 8066. The orientations of t [file pone.0078187.s001.pdf]

SUPPORTING INFORMATION

Complexes of neutralizing and non-neutralizing affinity matured Fabs with a mimetic of the internal trimeric coiled-coil of HIV-1 gp41

Elena Gustchina[1], Mi Li[2,3,], Rodolfo Ghirlando[4], Peter Schuck[5], John M. Louis[1], Jason Pierson[6], Prashant Rao[7], Sriram Subramaniam[7], Alla Gustchina[2]*, G. Marius Clore[1]*, and Alexander Wlodawer[2]*

[1]Laboratory of Chemical Physics, Building 5, National Institute of Diabetes and Digestive and Kidney Diseases, National Institutes of Health, Bethesda, MD 20892-0520

[2]Macromolecular Crystallography Laboratory, National Cancer Institute, Frederick, Maryland 21702-1201

[3]Basic Research Program, SAIC-Frederick, Frederick, MD 21702-1201

[4]Laboratory of Molecular Biology, National Institute of Diabetes and Digestive and Kidney Diseases, National Institutes of Health, Bethesda, MD 20892-0540

[5]Laboratory of Cellular Imaging and Macromolecular Biophysics, National Institute of Biomedical Imaging and Bioengineering, National Institutes of Health, Bethesda, MD 20892-5766

[6]FEI Company, Hillsboro, OR 97124

[7]Laboratory of Cell Biology, Center for Cancer Research, National Cancer Institute, Bethesda, MD 20892

Figure S1. SDS-PAGE of purified CCIZN36 disulfide-linked trimer under non-reducing and reducing conditions. Lane1, low molecular weight standards (GE Healthcare, Waukesha, WI), Lane 2, purified 3-H under non-reducing conditions (molecular weight 22674.8±22.8 Da verified using MALDI-TOF mass spectrometry), Lane 3, purified 3-H under reducing conditions (molecular weight of the monomer 7559.8±2.5 Da and dimer 15123.6±39.0 Da verified using MALDI-TOF mass spectrometry).

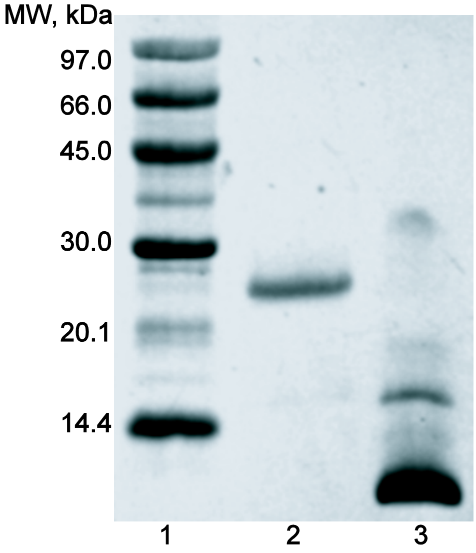

Figure S2. Fluorescence signal increments. 325 and 355 nm fluorescence emission intensity differences for (A) the 3-H and (B) the 8062 (blue) and 8066 (red) antibodies as a function of loading concentration. Fluorescence signal increments, $SI_{325\text{-}355}$ of -2.52 x 10^{11} M^{-1}, 2.68 x 10^{10} M^{-1} and 1.12 x 10^{11} M^{-1} are determined for the 3-H, 8062 and 8066 antibodies, respectively.

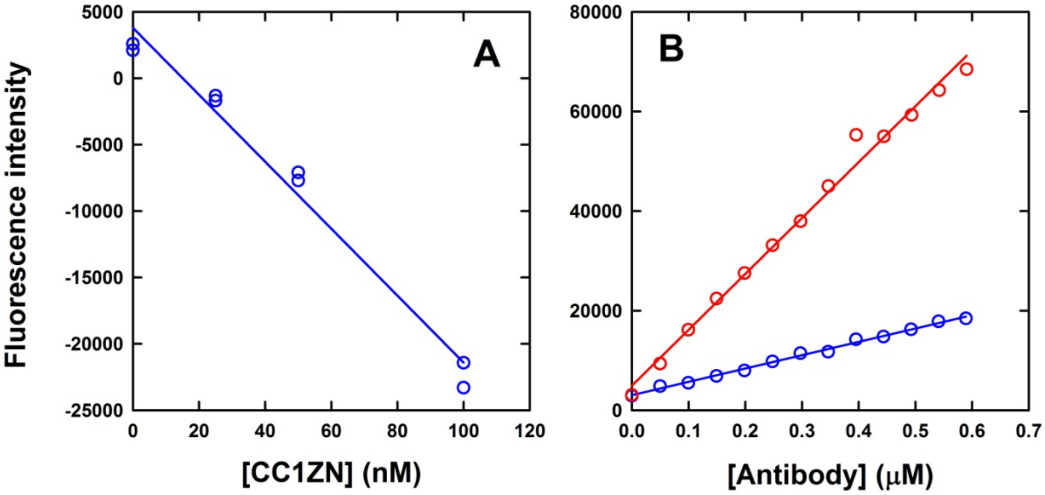

Figure S3 Crystal packing of the (Fab)$_3$/3-H complexes. (A) Complexes of Fab 8066 are aligned "head to tail". Each asymmetric unit contains one Fab and one N-HR helix (shown in different colors). (B) Helices of 3-H trimers form an infinite helix in the crystal. Hydrogen bonds between different 3-H trimers are shown in black.

A

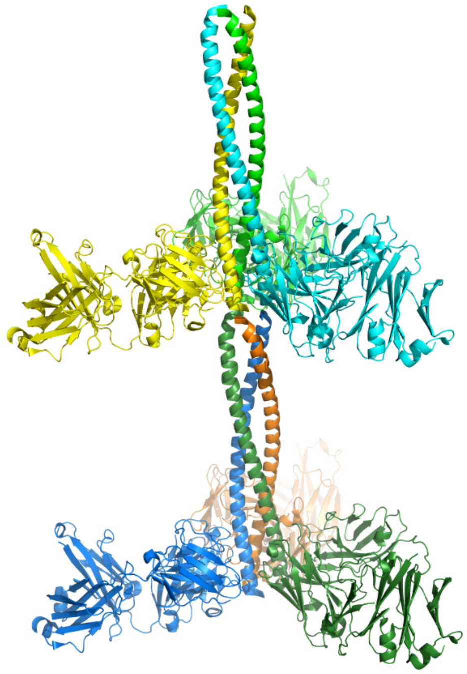

B

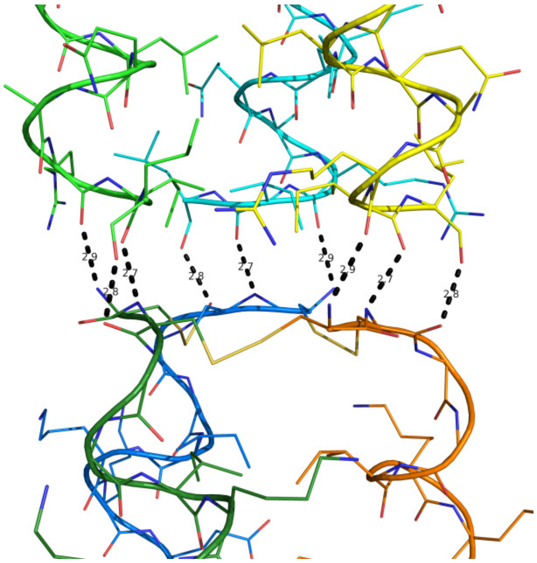

Figure S4. Superposition of the (Fab 8066)$_3$/3-H complex (red) and (Fab 8062)$_3$/3-H complex (blue). The superposition was based on Cα atoms of a β-sheet framework of the variable domain of a single Fab.

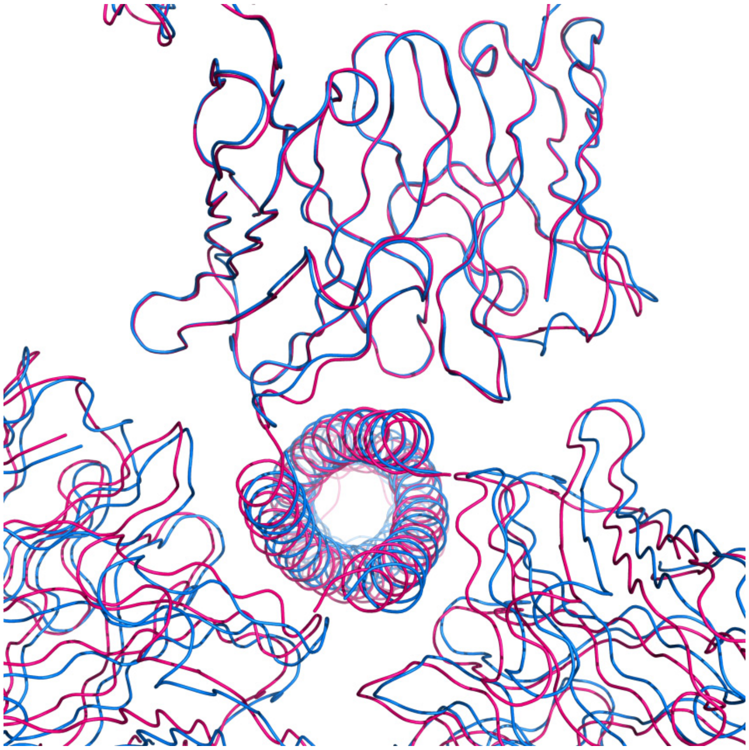

Figure S5. Selected examples of a single projection molecular images. The putative occupancies of Fab 8066 bound to the gp41 trimer are 1 (A), 2 (B), or 3 (C). Projection views of the crystallographically determined structure of the gp41-8066 complex are shown to mimic the orientation of the selected molecular images. The molecular structures shown in panels A and B were generated by removing either two copies or one copy, respectively of the 8066 Fab fragment, while the structure shown in panel C is that of the intact trimer with the bound Fab 8066. The orientations of the complexes were adjusted manually to show the best agreement with the electron microscopic images.

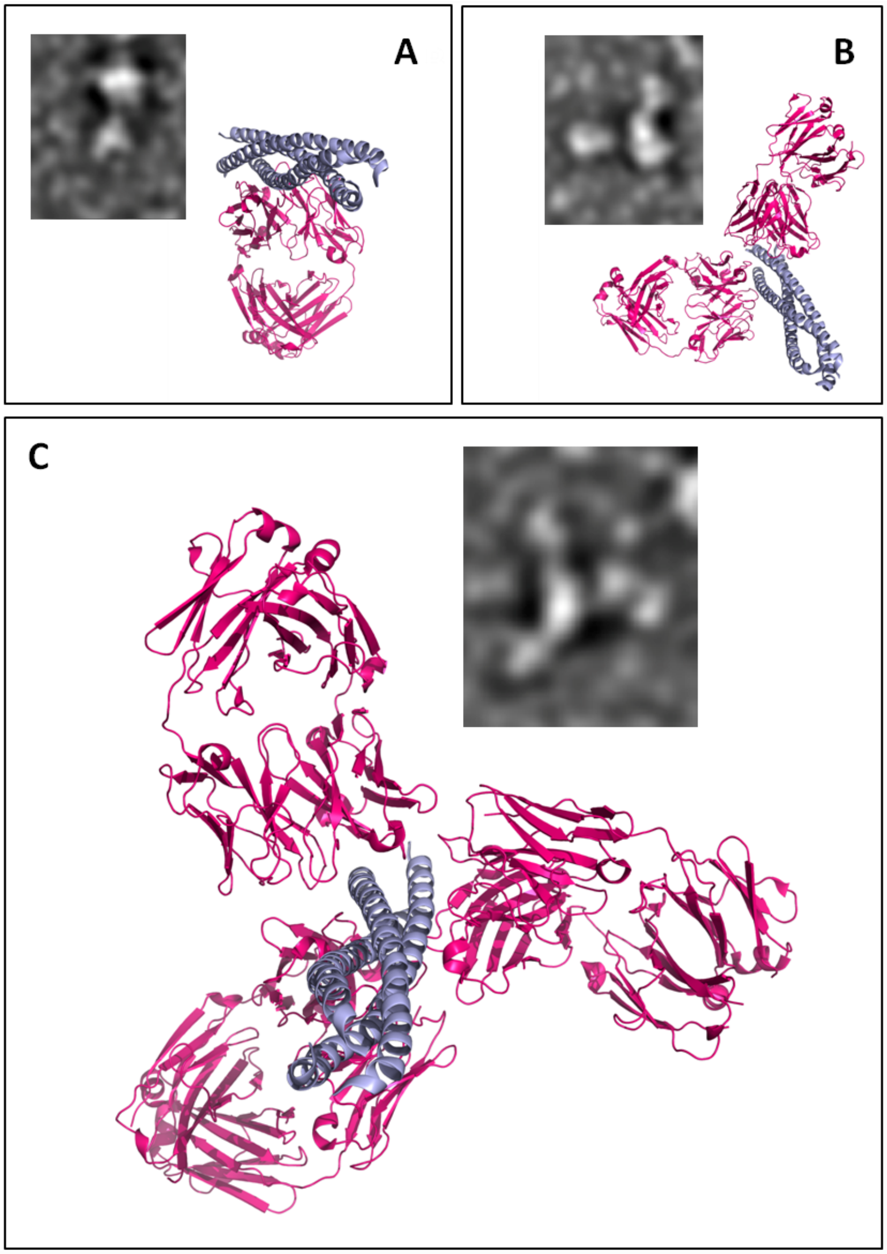

Table S1. Residue numbering of gp41 N-helices in 3 Fab/(CCIZN36)$_3$ complexes, Fab/5-Helix complexes and native full-length gp41. For the 5-Helix complex with Fab 8066 helices are highlighted with gray boxes and residues not visible in the electron density map are shown in small letters. For the 3 Fab/(CCIZN36)$_3$ complexes the helices are continuous and all residues are visible.

Sequence			*M*	*Q*	*L*	*L*	*S*	*G*	*I*	*V*	*Q*	*Q*	*Q*	*N*	*N*	*L*	*L*	*R*	*A*	*I*	*E*	*A*	*Q*	*Q*	*H*	*L*	*L*	*Q*	*L*	*T*	*V*	*W*	*G*	*I*	*K*	*Q*	*L*	*Q*	*A*	*R*	*I*	*L*	*A*	*G*	*g*	*s*	*g*	*g*
gp160		*No*	177	543	544	545	546	547	548	549	550	551	552	553	554	555	556	557	558	559	560	561	562	563	564	565	566	567	568	569	570	571	572	573	574	575	576	577	578	579	580	581	582					
3MA9	Na	No	1	2	3	4	5	6	7	8	9	10	11	12	13	14	15	16	17	18	19	20	21	22	23	24	25	26	27	28	29	30	31	32	33	34	35	36	37	38	39	40	41	42	43	44	45	46
4KHT	A	No					32	33	34	35	36	37	38	39	40	41	42	43	44	45	46	47	48	49	50	51	52	53	54	55	56	57	58	59	60	61	62	63	64	65	66	67						
Sequence			*H*	*T*	*T*	*W*	*M*	*E*	*W*	*D*	*R*	*E*	*I*	*N*	*N*	*Y*	*T*	*S*	*L*	*I*	*H*	*S*	*L*	*I*	*E*	*E*	*S*	*Q*	*N*	*Q*	*Q*	*E*	*K*	*N*	*E*	*Q*	*E*	*L*	*L*	*E*	*g*	*s*	*s*	*g*				
gp160		*No*	625	626	627	628	629	630	631	632	633	634	635	636	637	638	639	640	641	642	643	644	645	646	647	648	649	650	651	652	653	654	655	656	657	658	659	660	661	662								
3MA9	Ca	No	47	48	49	50	51	52	53	54	55	56	57	58	59	60	61	62	63	64	65	66	67	68	69	70	71	72	73	74	75	76	77	78	79	80	81	82	83	84	85	86	87	88				
Sequence			*G*	*Q*	*L*	*L*	*S*	*G*	*I*	*V*	*Q*	*Q*	*Q*	*N*	*N*	*L*	*L*	*R*	*A*	*I*	*E*	*A*	*Q*	*Q*	*H*	*L*	*L*	*Q*	*L*	*T*	*V*	*W*	*G*	*I*	*K*	*Q*	*L*	*Q*	*A*	*R*	*I*	*L*	*a*	*g*	*g*	*s*	*g*	*g*
gp160		*No*		543	544	545	546	547	548	549	550	551	552	553	554	555	556	557	558	559	560	561	562	563	564	565	566	567	568	569	570	571	572	573	574	575	576	577	578	579	580	581	582					
3MA9	Nb	No	89	90	91	92	93	94	95	96	97	98	99	100	101	102	103	104	105	106	107	108	109	110	111	112	113	114	115	116	117	118	119	120	121	122	123	124	125	126	127	128	129	130	131	132	133	134
4KHT	B	No					32	33	34	35	36	37	38	39	40	41	42	43	44	45	46	47	48	49	50	51	52	53	54	55	56	57	58	59	60	61	62	63	64	65	66	67						
Sequence			*h*	*T*	*T*	*W*	*M*	*E*	*W*	*D*	*R*	*E*	*I*	*N*	*N*	*Y*	*T*	*S*	*L*	*I*	*H*	*S*	*L*	*I*	*E*	*E*	*S*	*Q*	*N*	*Q*	*Q*	*E*	*K*	*N*	*E*	*Q*	*E*	*L*	*L*	*E*	*g*	*s*	*s*	*g*				
gp160		*No*	625	626	627	628	629	630	631	632	633	634	635	636	637	638	639	640	641	642	643	644	645	646	647	648	649	650	651	652	653	654	655	656	657	658	659	660	661	662								
3MA9	Cb	No		136	137	138	139	140	141	142	143	144	145	146	147	148	149	150	151	152	153	154	155	156	157	158	159	160	161	162	163	164	165	166	167	168	169	170	171	172	173	174	175	176				
Sequence			*G*	*Q*	*L*	*L*	*S*	*G*	*I*	*V*	*Q*	*Q*	*Q*	*N*	*N*	*L*	*L*	*R*	*A*	*I*	*E*	*A*	*Q*	*Q*	*H*	*L*	*L*	*Q*	*L*	*T*	*V*	*W*	*G*	*I*	*K*	*Q*	*L*	*Q*	*A*	*R*	*I*	*L*	*a*	*g*	*g*	*s*	*g*	*g*
gp160		*No*	[M]	543	544	545	546	547	548	549	550	551	552	553	554	555	556	557	558	559	560	561	562	563	564	565	566	567	568	569	570	571	572	573	574	575	576	577	578	579	580	581	582					
3MA9	Nc	No	177	178	179	180	181	182	183	184	185	186	187	188	189	190	191	192	193	194	195	196	197	198	199	200	201	202	203	204	205	206	207	208	209	210	211	212	213	214	215	216	217	218	219	220	221	222
4KHT	C	No					32	33	34	35	36	37	38	39	40	41	42	43	44	45	46	47	48	49	50	51	52	53	54	55	56	57	58	59	60	61	62	63	64	65	66	67						

Table S2. Antigen-antibody interactions, all with helix A (Na in 5-Helix), except where indicated, helix B is Nc in 5-Helix, C helix residues in italics, Hydrophobic contacts in bold, *Hydrogen bonds/polar contacts

AB Loop	AB Residue	8066/5-Helix		3 8066(/CCIZN36)$_3$		8062/5-Helix		3 8062/(CCIZN36)$_3$	
		CCP4	MolProbity	CCP4	MolProbity	CCP4	MolProbity	CCP4	MolProbity
H1	N30							**L568**	
	S31	**L568 x 5**	**L568**	**L568 x 3**	**L568 x 9**	**L568 x 2**	**L568**	**L568 x 2**	**L568 x 4**
				L568* x 3	L568* x 4	L568* x 2		L568*	
		**H564 x 5**		**H564 x 5**	**H564 x 5**	**H564**	**H564 x 2**	**H564 x 3**	**H564 x 5**
		H564* x 3	H564* x 2	H564* x 2	H564* x 2	H564* x 2	H564* x 2	H564* x 4	H564* x 4
	Y32			L568* x 2				L568* x 2	
	A33			**L568**	**L568 x 2**			**L568**	**L568**
H2	S50			Q575* x2	Q575* x2	Q575*		Q575* x3	Q575*
	I51	Q575*		Q575*					
	I52	**L568 x 2**	**L568 x 3**	**L568 x 4**	**L568 x 4**	**L568 x 4**	**L568 x 8**	**L568 x 4**	**L568 x 8**
		L568*		L568*	L568*			L568*	
		**W571 x 6**	**W571 x 2**	**W571 x 2**	**W571 x 2**	**W571**		**W571 x 4**	**W571 x 4**
					**G572 x 2**				
		**Q575**	**Q575**	**Q575 x 2**		**Q575**		**Q575**	
		Q575* x 4	Q575* x 3	Q575* x 3	Q575* x 3			Q575*	Q575*
	I/L53	**L565**		**L565**	**L565 x 2**	**L565**	**L565 x 2**		**L565 x 5**
		**L568**				**L568**	**L568 x 2**	**L568**	**L568**
		**V570 x 3(helixB)**	**V570 x 5(helixB)**	**V570 x 4(helixB)**		**V570 x 4(helixB)**	**V570 x 2(helixB)**	**V570 x 3(helixB)**	**V570 x 4(helixB)**
		**K574 x 3(helixB)**		**K574 x 2(helixB)**		**K574 (helixB)**		**K574 (helixB)**	
		K574* x 4(helixB)	K574* x 3(helixB)	K574* x 2(helixB)	K574* x 6(helixB)	K574* (helixB)	K574* x 2(helixB)	K574* x2(helixB)	K574* x5(helixB)
	F54			**L565**					
		**L568 x 4**	**L568 x 3**	**L568 x 2**		**L568 x 4**	**L568 x 2**	**L568 x 3**	

	L568*	L568* x 4	L568* x 2	L568*	L568* x 2	L568* x 5	L568* x 2	L568* x 3
	**T569 x 5**	**T569 x 10**	**T569 x 6**	**T569 x 9**	**T569 x 3**	**T569 x 10**	**T569 x 7**	**T569 x 8**
	**V570 x 8(helixB)**	**V570 x 12(helixB)**	**V570 x 10(helixB)**	**V570 x 16(helixB)**	**V570 x 5(helixB)**	**V570 (helixB)**	**V570 x 6(helixB)**	**V570 x 4(helixB)**
			V570* (helixB)		V570* (helixB)		V570* (helixB)	
	**G572**	**G572 x 2**	**G572 x 2**	**G572**	**G572 x 2**	**G572**	**G572 x 2**	
	**I573 x 14(helixB)**	**I573 x 20(helixB)**	**I573 x 14(helixB)**	**I573 x 19(helixB)**	**I573 x 13(helixB)**	**I573 x 6(helixB)**	**I573 x 9(helixB)**	**I573 x 4(helixB)**
	**K574 x 6(helixB)**	**K574 x 5(helixB)**	**K574 x 3(helixB)**	**K574 (helixB)**	**K574 x 4(helixB)**	**K574 x 7(helixB)**	**K574 x 2(helixB)**	
	K574* (helixB)		K574* (helixB)		K574* (helixB)		K574* x 2(helixB)	
	**Q577 x 2(helixB)**		**Q577 (helixB)**		**Q577 x 2(helixB)**		**Q577 (helixB)**	
	Q577* x 2(helixB)	Q577* x 2(helixB)	Q577* x 2(helixB)	Q577* x 2(helixB)	Q577* x 3(helixB)	Q577* x 3(helixB)	Q577* x 3(helixB)	Q577* (helixB)
G55	**Q577 x 3(helixB)**	**Q577 x 2(helixB)**	**Q577 x 2(helixB)**		**Q577 (helixB)**		**Q577 (helixB)**	
	Q577* x 3(helixB)	Q577* x 3(helixB)	Q577* x 3(helixB)	Q577* x 3(helixB)	Q577* (helixB)		Q577* x 2(helixB)	
T/F56	**G572 x 3**	**G572**	**G572**		**G572 x 4**	**G572 x 2**	**G572**	
	G572* x 4	G572*	G572* x 3		G572*	G572* x 4	G572* x 2	G572* x 2
	**Q575 x 6**	**Q575 x 5**	**Q575 x 2**		**Q575 x 8**	**Q575 x 12**	**Q575 x 2**	
	Q575* x 7	Q575* x 8	Q575* x 2	Q575* x 5	Q575* x 5	Q575* x 3	Q575* x 3	
	**L576 x 3**	**L576 x 6**	**L576 x 2**	**L576 x 2**	**L576 x 11**	**L576 x 8**	**L576 x 12**	**L576 x 4**
	**I573 (helixB)**		**I573 (helixB)**					
	**Q577 x 6(helixB)**	**Q577 x 7(helixB)**	**Q577 x 7(helixB)**	**Q577 x 5(helixB)**	**Q577 x 9(helixB)**	**Q577 x 6(helixB)**	**Q577 x 11(helixB)**	**Q577 x 3(helixB)**
	Q577* x 3(helixB)	Q577* x 5(helixB)	Q577* x 8(helixB)	Q577* x 2(helixB)	Q577* x 2(helixB)	Q577* x 2(helixB)	Q577* (helixB)	Q577* x 2(helixB)
					**I580 (helixB)**			
							**L581 x 3(helixB)**	**L581 x 8(helixB)**

	T/V57	**Q575 x 6**		**Q575 x 3**	**Q575 x 2**				
		Q575* x 6	Q575* x 6	Q575* x 6	Q575* x 6		Q575* x 2		
		**R579**	**R579 x 2**	**R579 x 2**				**R579**	
		R579* x 4	R579* x 8	R579* x 5	R579* x 3	R579* x 3		R579* x 5	R579* x 10
		Q577* (helixB)		L581* (helixB)					
	N/V58	W571*							
		**Q575 x 5**	**Q575 x 3**	**Q575 x 6**	**Q575 x 10**	**Q575 x 10**	**Q575 x 8**	**Q575 x 8**	**Q575 x 12**
		Q575*	Q575* x 4	Q575* x 6	Q575* x 11	Q575* x 2		Q575* x 2	
		R579*		**R579**				**R579 x 3**	**R579 x 2**
	Y59			**R579 x 2**	**R579 x 7**			**R579 x 2**	
				R579* x 2	R579* x 4				
	Q64				**R579**	**R579 x 3**			
H3	Y95	**W571 x 7**	**W571 x 8**	**W571 x 4**	**W571 x 3**	**W571 x 4**	**W571 x 4**	**W571 x 2**	
		W571* x 3	W571* x 2	W571* x 3	W571* x 3	W571* x 2	W571* x 5	W571* x 2	W571*
	F96	**H564 x 4**	**H564 x 2**	**H564**	**H564 x 2**	**H564 x 3**	**H564 x 2**	**H564 x 3**	**H564 x 3**
		H564*	H564* x 3	H564*	H564* x 3	H564*	H564* x 4	H564*	H564* x 2
		**Q567 x 8**	**Q567 x 9**	**Q567 x 6**	**Q567 x 2**	**Q567 x 4**	**Q567 x 10**	**Q567 x 3**	**Q567 x 8**
		Q567* x 2		Q567*		Q567* x 2		Q567* x 2	
		**L568 x 4**	**L568 x 5**	**L568 x 4**	**L568 x 3**	**L568 x 4**	**L568**	**L568 x 4**	
		**W571 x 9**	**W571 x 9**	**W571 x 12**	**W571 x 6**	**W571 x 13**	**W571 x 13**	**W571 x 14**	**W571 x 8**
	D97	**H564**				**H564**		**H564**	
		H564* x 2		H564* x 2		H564*		H564* x 2	
	T98	**H564 x 2**		**H564 x 2**		**H564**		**H564**	
		H564* x 2		H564* x 3		H564* x 2		H564* x 2	
	Y99	**E560 x 7**				**E560 x 8**	**E560**		
		E560* x 2				E560*			
		**Q563 x 11**	**Q563 x 15**	**Q563 x 10**	**Q563 x 4**	**Q563 x 7**	**Q563 x 3**	**Q563 x 10**	
		Q563*		Q563* x 6	Q563* x 7	Q563*		Q563* x 5	Q563*
		**H564 x 5**	**H564 x 11**	**H564 x 3**	**H564 x 3**	**H564 x 5**	**H564 x 5**	**H564**	
			H564* x 4	H564*			H564* x 2		
		**Q567 x 2**		**Q567 x 9**	**Q567 x 6**			**Q567 x 4**	

		Q567* x 3	Q567* x 3	Q567* x 2	Q567* x 4	Q567* x 2	Q567* x 3	Q567* x 3	Q567* x 4
		H643					***H643 x 3***		
			_H643* x 7_				_H643*_		
		I646	***I646 x 13***			***I646***	***I646 x 5***		
			_I646* x 6_				_I646* x 6_		
	N100	Q567*							
							H643 x 4		
			_H643*_						
	Y100B	**Q567 x 4**				**Q567 x 3**	**Q567**	**Q567 x 3**	**Q567 x 3**
		Q567*		Q567* x 2		Q567* x 2	Q567* x 3	Q567*	Q567* x 8
		**W571 x 4**	**W571 x 4**	**W571 x 5**	**W571 x 10**	**W571 x 6**	**W571 x 4**	**W571 x 9**	**W571 x 6**
			_T639*_				_T639*_		
							_H643*_		
L1	P29	***N636***	***N636 x 8***			***N636***			
			_N636 x 6_						
	E31	***S640***							
			_S640*_						
						H643	***H643 x 2***		
							_H643* x 3_		
	Y32	**W571 x 4**	**W571 x 5**	**W571 x 3**		**W571 x 3**		**W571 x 6**	**W571 x 5**
				W571*				W571* x 2	
					**K574 x 2**				**K574 x 2**
		K574*		K574* x 2	K574* x 2			K574* x 2	K574* x 2
		N636				***N636***			
			_N636*_						
L3	W91	**W571 x 14**	**W571 x 20**	**W571 x 14**	**W571 x 10**	**W571 x 13**	**W571 x 7**	**W571 x 11**	**W571 x 11**
	S93	**K574 x 2**							
		K574* x 4	K574* x 6	K574*		K574* x 2	K574*	K574* x 2	K574* x 2
		D632 x 2	***D632 x 2***			***D632 x 2***			
			_D632* x 4_						

	N636							
		N636 x 2*						
M94	**K574 x 5**	**K574 x 3**						
	K574* x 3				K574* x 4	K574*		
							Q577* x 3	
			**A578**					
		W628 x 2						
		*W628**						
	M629	***M629 x 4***				***M629 x 10***		
		M629 x 5*				*M629**		
		W631 x 2						
	D632 x 2	***D632 x 14***			***D632 x 2***	***D632 x 4***		
		D632 x 2*				*D632**		
T95			**A578 x 2**		**A578 x 3**	**A578 x 3**	**A578 x 3**	**A578 x 3**
					A578*		A578*	A578* x 2
V95A	**W571 x 18**	**W571 x 22**	**W571 x 6**	**W571 x 12**	**W571 x 12**	**W571 x 19**	**W571 x 3**	**W571 x 7**
	W571* x 4	W571* x 2	W571*		W571* x 2			
	**K574 x 4**	**K574 x 5**	**K574 x 5**	**K574 x 4**	**K574 x 5**	**K574 x 5**	**K574 x 7**	**K574 x 6**
			K574*		K574*		K574*	K574*
	**Q575**	**Q575 x 2**	**Q575 x 3**	**Q575 x 8**	**Q575 x 3**	**Q575 x 11**	**Q575 x 5**	**Q575 x 9**
			**A578**				**A578**	

Table S3. Antibody-antibody contacts in 3 8066/(CCIZN36)$_3$ and 3 8062/(CCIZN36)$_3$. Hydrophobic contacts in bold, *Hydrogen bonds/polar contacts

3 8066(/CCIZN36)$_3$			3 8062/(CCIZN36)$_3$	
Loop 71-77	CDR-L3	CDR-L1	Loop 71-77	CDR-L1
A71	M94* x 2			
			D72	N27*
E73	S93* x 3		E73	P29*
				**Y30 x 2**
				Y30* x 4
S74		**I28 x 3**	S74	**N27 x 2**
		I28* x 2		N27* x 3
		D27*		

Movie S1. Structural rearrangement of the N-HR trimer required for (Fab 8062)$_3$/3-H complex formation is illustrated using morphing of the structures from the N-HR trimer from the 6-Helix bundle (PDB ID: 1env, yellow) to the N-HR trimer from the (Fab 8062)$_3$/3-H complex (green). The N trimer from the (Fab 8066)$_3$/3-H complex (red) is used as a reference. C-HR helices of the 6-Helix bundle are shown in gray. These changes in the orientation of N-HR trimer upon binding of three Fab 8062 molecules lead to collisions between the side chains of N-HR trimer (green) and the C-HR helices as present in the original 6-Helix bundle structure (gray).
